# Genetic Variability among Complete Human Respiratory Syncytial Virus Subgroup A Genomes: Bridging Molecular Evolutionary Dynamics and Epidemiology

**DOI:** 10.1371/journal.pone.0051439

**Published:** 2012-12-07

**Authors:** Lydia Tan, Philippe Lemey, Lieselot Houspie, Marco C. Viveen, Nicolaas J. G. Jansen, Anton M. van Loon, Emmanuel Wiertz, Grada M. van Bleek, Darren P. Martin, Frank E. Coenjaerts

**Affiliations:** 1 Department of Medical Microbiology, University Medical Center Utrecht, Utrecht, The Netherlands; 2 Rega Institute for Medical Research, Department of Microbiology and Immunology, KU Leuven, Leuven, Belgium; 3 Department of Pediatrics, Wilhelmina Children’s Hospital, University Medical Center Utrecht, Utrecht, The Netherlands; 4 Institute of Infectious Diseases and Molecular Medicine, Computational Biology Department, Faculty of Health Sciences, University of Cape Town, Cape Town, South Africa; University of Hong Kong, China

## Abstract

Human respiratory syncytial virus (RSV) is an important cause of severe lower respiratory tract infections in infants and the elderly. In the vast majority of cases, however, RSV infections run mild and symptoms resemble those of a common cold. The immunological, clinical, and epidemiological profile of severe RSV infections suggests a disease caused by a virus with typical seasonal transmission behavior, lacking clear-cut virulence factors, but instead causing disease by modifying the host’s immune response in a way that stimulates pathogenesis. Yet, the interplay between RSV-evoked immune responses and epidemic behavior, and how this affects the genomic evolutionary dynamics of the virus, remains poorly understood. Here, we present a comprehensive collection of 33 novel RSV subgroup A genomes from strains sampled over the last decade, and provide the first measurement of RSV-A genomic diversity through time in a phylodynamic framework. In addition, we map amino acid substitutions per protein to determine mutational hotspots in specific domains. Using Bayesian genealogical inference, we estimated the genomic evolutionary rate to be 6.47×10^−4^ (credible interval: 5.56×10^−4^, 7.38×10^−4^) substitutions/site/year, considerably slower than previous estimates based on G gene sequences only. The G gene is however marked by elevated substitution rates compared to other RSV genes, which can be attributed to relaxed selective constraints. In line with this, site-specific selection analyses identify the G gene as the major target of diversifying selection. Importantly, statistical analysis demonstrates that the immune driven positive selection does not leave a measurable imprint on the genome phylogeny, implying that RSV lineage replacement mainly follows nonselective epidemiological processes. The roughly 50 years of RSV-A genomic evolution are characterized by a constant population size through time and general co-circulation of lineages over many epidemic seasons – a conclusion that might be taken into account when developing future therapeutic and preventive strategies.

## Introduction

Human respiratory syncytial virus (RSV) is the single most important cause of severe lower respiratory tract infections (LRTI) in infants and young children. As a consequence, RSV infections are the most frequent cause of hospitalization of infants and young children in industrialized countries. For example, RSV is responsible for at least 100,000 infant hospitalizations for pneumonia or bronchiolitis every year in the USA alone [1]. The RSV disease spectrum ranges from mild symptoms such as rhinitis and otitis media, to severe illness such as bronchiolitis or pneumonia, which require supportive care such as mechanical ventilation [2]. By the age of two, most children have been infected with RSV, with about half having experienced two or more infections [3]. Elderly people, patients suffering from cardiopulmonary diseases and immuno-compromised individuals also are at risk for severe RSV disease [4], [5], [6]. RSV attack rates in nursing homes in the USA are approximately 5–10% per year with a 2–8% case fatality rate, amounting to 10,000 deaths per year among persons above 64 years of age [4]. Among elderly persons followed for 3 consecutive winters, RSV infection accounted for 10.6% of hospitalizations for pneumonia, 11.4% of hospitalizations for obstructive pulmonary disease, 5.4% for congestive heart failure, and 7.2% for asthma [7].

RSV is classified in the genus *Pneumovirus* belonging to the *Paramyxoviridae* family and has an enveloped, non-segmented, single-stranded, negative sense RNA genome of approximately 15,000 nucleotides. The virus has 11 genes coding for non-structural proteins 1 and 2 (NS1 and -2), the nucleocapsid protein (N), phosphoprotein (P), matrix protein (M), small hydrophobic protein (SH), attachment glycoprotein (G), fusion glycoprotein (F), transcription regulatory proteins M2-1 and M2-2 and, finally, a large polymerase (L).

In Europe and North America, RSV disease occurs as well-defined seasonal epidemic outbreaks during the winter and spring months. On the other hand, studies in tropical countries have often reported an increase in RSV during the rainy season, but this has not been a consistent finding [8]. The exact factors responsible for the typical RSV transmission dynamics are not well understood. Multiple genotypes can be present in a single population while new genotypes may replace older predominating genotypes over successive epidemic seasons. Among different populations, variation in herd immunity has been suggested to play a role in the observed genotypic distribution patterns [9], [10], [11]. Community-specific cultural and behavioral patterns might also affect the acquisition and spread of RSV infection.

Antibody cross-reactivity patterns have led to the identification of two antigenic subgroups (A and B) for RSV and these subgroups have been further classified into genotypes based on genetic divergence within the G gene [12], [13], [14]. Subgroup A generally dominates during epidemics and subgroup B is mostly involved in re-infection, although both can co-circulate [15], [16], [17]. Re-infections with RSV occur frequently throughout life and it has been suggested that the evasion of pre-existing host immune responses is particularly facilitated by antigenic variability of the G protein both between and within the two major antigenic subgroups [18], [19].

Development of vaccines to prevent RSV infection has been complicated by several factors, including the risk of potentiating naturally occurring disease. Early attempts at vaccinating children in the 1960s with a formalin-inactivated RSV vaccine showed that vaccinated children suffered from more severe disease on subsequent exposure to the virus as compared to the control-vaccinated group [20], [21], [22], [23]. The enhanced severity of disease has been reproduced in animal models and is thought to result from inadequate levels of serum-neutralizing antibodies, antibody-mediated CD4(+) memory T cell response enhancement, and infection mediated CD8(+) memory T cell suppression [24], [25], [26], [27]. Live, attenuated RSV vaccines have apparently not exacerbated pulmonary disease upon natural infection, but have also failed to provide any appreciable degree of protection [28], [29], [30]. On the basis of these and other observations, it has been hypothesized that the development of disease in RSV infected individuals is principally caused by immune responses induced by the virus [31], [32].

From the clinical, immunological and epidemiological profile of RSV a picture emerges of a severe disease that is caused by a virus with typical seasonal transmission behavior, no clear-cut virulence factors, and the capacity to modify host immune responses in a way that affects pathogenesis (e.g. through interfering with Toll-like receptor-signaling [33]). However, despite its potentially important impact on global patterns of RSV genetic diversity, the interplay between RSV induced immune responses and the epidemiological and evolutionary dynamics of RSV remain poorly understood.

Phylodynamic analyses of viral genetic data are increasingly being used to illuminate how evolutionary and ecological processes can jointly drive fluctuations in the genetic diversity of viral populations [34]. Such analyses hold the potential to reveal viral population dynamics based on phylogenetic patterns, to determine the likely short- and long-term evolutionary impacts of mutations that enable viruses to escape host immune responses, and to describe how these factors might collectively influence the epidemic behavior of viruses. Although the molecular epidemiology and evolutionary dynamics of RSV have been actively studied in the past, much of our current knowledge in this area has been determined through the analysis of single genes. Little is known on how the evolutionary patterns of these individual genes – and in particular the most actively studied G gene – reflect the genomic dynamics of RSV. Complete genome sequences are only starting to be generated for isolates of this species [35], [36], but such efforts may become more common with the widespread use of next generation sequencing technologies. As recently pointed out for viruses in general [37], generating RSV genomes is only the first step on the path to understanding the processes that shape the epidemiology and evolution of this virus. Here, we present analyses of the most comprehensive collection of RSV-A complete genomes to date, including thirty three strains sampled over the last decade, and provide the first measurements made within a phylodynamic framework of RSV-A genomic diversity through time. In addition to detailed analyses of genome-wide variability we present estimates of key evolutionary parameters such as the genomic rate of evolution. In particular we demonstrate that relative to the remainder of the RSV genome, the G gene has a larger number of sites evolving under diversifying selection and is, probably as a consequence of this, the fastest evolving RSV-A gene. Finally, we test the degrees to which host immune responses have impacted both patterns of selection detectable within RSV genomes and global-scale RSV population dynamics.

## Materials and Methods

### Ethics Statement

This study was performed according to the guidelines of the institutional ethics committees (METC for Dutch samples; CME for Belgian samples) and in concordance with Dutch privacy legislation. All anonymized clinical strains originated from the routine diagnostic process prior to the current study. The institutional review board (IRB) confirmed (protocol 12/320) that viral strains are not regarded as patient-owned material and consequently the use of these strains is not restricted in the applicable Dutch law (“Law Medical Scientific Research with People”, WMO; art. 1b).

### Viral Isolates

Nasopharyngeal swabs and tracheal aspirates were collected between 2001 and 2011 from children ranging in age from 2 weeks to 5 years, who were hospitalized with respiratory distress symptoms on the pediatric wards of the Wilhelmina Children’s Hospital in Utrecht, The Netherlands or the Gasthuisberg University Hospital of Leuven, Belgium (Table S1). Collected samples were cultured immediately and checked for the formation of syncytia in HEp2 cell cultures, a typical RSV infection phenotype. Those exhibiting RSV specific cytopathological effects (CPE) and testing positive for RSV by real-time PCR, were harvested and stored at −80°C.

### Viral RNA Extraction, cDNA Synthesis and Real-time Taqman PCR

RSV strains were routinely cultured on HEp2 cells for 2 passages at 33°C. Virus was harvested from cultures that exhibited CPE in 80% of cells and viral genomic RNA was isolated using a MagnaPure LC total nucleic acid kit (Roche Diagnostics, Mannheim, Germany). The isolated viral RNA was reverse transcribed using a MultiScribe reverse transcriptase kit and random hexamers (Applied Biosystems, Foster City, CA), according to the manufacturer’s guidelines. Primers and probes designed on the basis of highly conserved genomic regions of the N gene for both RSV subgroup A (RSV-A) and B (RSV-B) were used for subtyping of the RSV patient strains. The following primers and probes were used:

RSA-1∶5′-AGATCAACTTCTGTCATCCAGCAA-3′

RSA-2∶5′-TTCTGCACATCATAATTAGGAGTATCAAT-3′

RSB-1∶5′-AAGATGCAAATCATAAATTCACAGGA-3′

RSB-2∶5′-TGATATCCAGCATCTTTAAGTATCTTTATAGTG-3′

RSA probe: 5′-CACCATCCAACGGAGCACAGGAGAT-3′

RSB probe: 5′-TTCCCTTCCTAACCTGGACATAGCATATAACATACCT-3′

Murine encephalomyocarditis virus was used as an internal control. Samples were assayed in a 25 µl reaction mixture containing 10 µl of cDNA, TaqMan universal PCR master mix (Applied Biosystems, ABI), primers (900 nM RSV-A primers and 300 nM RSV-B primers), and fluorogenic probes (58.3 nM RSV-A probe and 66.7 nM RSV-B probe) labeled with the 5′ reporter dye 6-carboxy-fluorescein (FAM) and the 3′ quencher dye 6-carboxy-tetramethyl-rhodamine (TAMRA). Amplification and detection were performed with an ABI 7900 HT system for 2 min at 50°C, 10 min at 95°C, and 45 cycles of 15 sec at 95°C and 1 min at 60°C. Samples were controlled for the presence of possible inhibitors of the amplification reaction by the indicated internal control, signals of which had to range within a clear-cut interval.

### Genomic RSV cDNA Synthesis and Sequencing

For sequencing purposes, human RSV PCR fragments were obtained by fractional amplification of MagNAPure LC genomic RNA isolates using the Superscript III one-step RT-PCR System with Platinum Taq High Fidelity kit (Invitrogen) and a 9800 Fast thermal cycler (ABI) according to the manufacturers protocol. PCR products were applied to a 1% agarose gel and purified from the gel by Qiaquick spin columns (Qiagen). Isolated fragments were used for whole genome sequencing.

The majority of the listed RSV strains (Table S1) was sequenced according to the whole genome sequence protocol recently described by Kumaria *et al* [35] using the conventional Sanger technique. Fragments ranging between 650 and 1,400 nucleotides were sequenced on an ABI 3730 48-capillary DNA analyzer using Big-Dye Terminator 3.1 (ABI). Table S2 provides an overview of the primers used. Indicated strains (Table S1) were (partly) sequenced via commercial 454-sequencing (Keygene, Wageningen, The Netherlands). The resulting sequence information was assembled into RSV whole genome sequences through alignment with the reference RSV A2 strain (M74568) using Seqman software (DNASTAR lasergene 8).

### Protein Substitution Analysis

Gene sequences were extracted from the whole genome sequences and the consensus of each gene was derived from the set of patient and reference strains using Seqman software. Gene sequences were translated into protein coding sequences using Seaview4 and subsequently aligned via the EMBL-EBI ClustalW2-Multiple Sequence Alignment tool. For each strain, sequence variability scores were calculated per gene relative to the consensus sequence for all clinical and reference strains. Protein substitutions per site were mapped using the program Plot0.997. For the RSV fusion protein, substitutions were marked in the 3RKI_PDG crystal structure with MacPyMOL (available at http://www.pymol.org/pymol). The NetNGlyc 1.0 server [38] was used to predict the gain and loss of N-glycosylation sites.

### Data Set Compilation and Recombination Analysis

To study the complete genome evolutionary dynamics of RSV, we combined our newly obtained complete genomes with the genomes from the recent studies of Kumaria *et al.* (2011) and Rebuffo-Scheer *et al.* (2011). The latter (JF920046-JF920070), were recovered from nasopharyngeal and nasal swabs collected from patients in the Milwaukee metro area. A total of 72 RSV-A complete genomes were aligned using Mafft [39] and manually edited in Se-Al (available at http://tree.bio.ed.ac.uk/software/seal/). Recombination was analyzed using the RDP, GENECONV, RECSCAN, MAXCHI, CHIMAERA, SISCAN, and 3SEQ recombination detection methods implemented in RDP3 [40], [41]. Only potential recombination events detected by two or more of these methods, together with phylogenetic evidence of recombination, were considered robust evidence of likely recombination. Based on the considerable recombination signal detected in the complete genomes obtained by Kumaria *et al*. (2011), further analyses were performed without these 14 sequences (on an alignment of 58 sequences). Since six recombination events evident within five of the remaining sequences may potentially undermine the validity of any phylogeny based analysis (the evolutionary history of recombinant sequences can in many cases not be adequately described by a single phylogenetic tree), we took the necessary steps to appropriately account for these recombination events. We noted that the five recombinant genomes all had a clear majority of their sequences inherited from a single major parent and a minority of sequences inherited from one or more minor parents (Fig. S1). We pursued two strategies to account for these recombination events. In the first strategy we simply discarded the portion of recombinant sequences that had been inherited from their minor parents (corresponding to the B and C regions in Figure S1) and replaced these “deleted” genomic sites in the five aligned recombinant sequences with gap characters (i.e. we treated these sites as though they are unobserved in the probabilistic inferences). In the second strategy rather than simply deleting the genomic sites apparently derived from the minor parent, we separated the genomic regions of the recombinants derived from their major and minor parents and treated these separated regions as though they were separately sampled sequences. Importantly, a single phylogenetic history could be safely assumed for the genomic sequence datasets yielded by both strategies. For the selection pressure analyses of the complete genome, we stripped the non-coding regions from the alignments and extracted a concatenated coding gene alignment. To scrutinize the evolutionary dynamics of the G gene, we also aligned the G gene sequences of our complete genomes to a data set previously analyzed by Zlateva *et al*. (2004; Table S3), resulting in an alignment of 185 sequences encompassing amino acid position 91 to 297 of the G protein.

### Bayesian Estimation of RSV Evolutionary History

To evaluate whether our complete genome data set showed a clear signal of nucleotide divergence throughout the sampling time interval, we explored linear regressions of root-to-tip divergence as a function of sampling time using Path-O-Gen (available at http://tree.bio.ed.ac.uk/software/pathogen/) [42]. For this purpose, we employed maximum likelihood trees reconstructed using PhyML [43] based on a general time-reversible (GTR) substitution model and a discretized gamma distribution to model rate variation among sites. The exact sampling data was known for all genomes except for six sequences obtained from Rebuffo-Scheer *et al.* (2011), which were set at the midpoint of the reported sampling year for this exploratory analysis.

We reconstructed the RSV evolutionary and demographic history using Bayesian Markov chain Monte Carlo (MCMC) in BEAST v1.7 [44]. BEAST infers a posterior distribution of time-measured genealogies based on a full probabilistic model including a molecular clock model, a nucleotide substitution model and a coalescent model as prior distribution for the tree. We used a general time-reversible (GTR) substitution model and a discretized gamma distribution to model rate variation among sites. To examine substitution rate variability across the genome, we also applied a partition model that allowed for separate substitution rates for the different genes and the single non-coding genome region. The fit of strict and relaxed clock models, assuming homogenous and heterogeneous substitution rates across the branches of the phylogeny respectively [45], was assessed based on a range of statistics including marginal likelihoods estimated using the harmonic mean, Akaike's information criterion through MCMC (AICM), path sampling (PS) estimators and stepping-stone sampling (SS) estimators [46], [47]. For the six sequences for which the exact sampling date was not available, we estimated the tip ages constraining them within a time interval of one year (using a uniform prior, [48]). We used the Bayesian skyline plot model as a flexible demographic prior allowing us to reconstruct how the effective population size changed through time [49]. MCMC analyses were run until convergence could be safely assumed, as evaluated using Tracer (available at http://tree.bio.ed.ac.uk/software/tracer/). Marginal posterior distributions for the evolutionary rate and divergence times, such as the times to most recent common ancestors (TMRCA) of various groups of sequences, were summarized using means and 95% highest posterior density intervals (HPDs). We represented the posterior tree distribution using a maximum clade credibility (MCC) tree annotated with divergence time and evolutionary rate summaries and provided a visualization of this tree using FigTree (http://tree.bio.ed.ac.uk/software/figtree/) [45].

To test whether the phylogenetic tree shape in the posterior distribution deviated from neutral expectations, we performed a genealogical test based on posterior predictive simulation [50]. Briefly, we summarize the tree shapes obtained by our Bayesian coalescent analysis using the genealogical Fu and Li statistic (*D_F_*). This statistic compares the length of terminal branches to the total length of the coalescent genealogy and returns negative values for long terminal branch lengths, which in turn indicates an excess of slightly deleterious mutations on these branches and hence a deviation from neutrality. The posterior predictive simulation randomly simulates trees with the same number of tips, the same tip ages, and under the same neutral coalescent model as the one applied to the real data. We employed the posterior predictive simulation extension that allows simulation of trees under the Bayesian skyline plot model [51]. Finally, we derive *P*-values for this genealogical neutrality test based on the frequency at which the *D_F_* of the inferred trees was more extreme than the *D_F_* of the simulated trees.

### Selective Pressure Analyses

For the concatenated coding genes, we identified sites under diversifying selection using a fixed-effects likelihood (FEL) approach [52] and a recently developed renaissance counting (RC) procedure [53]. The FEL method fits codon models to each site independently and performs a likelihood ratio test to evaluate whether a model that assumes equal non-synonymous and synonymous rates (*d*_N_ = *d*_S_) can be rejected in favor of a model that allows for different *d*_N_ and *d*_S_ rates. The RC approach combines stochastic mapping under nucleotide substitution models and empirical Bayes to estimate site-specific *d*_N_/*d*_S_ ratios, and quantifies their uncertainty while accommodating phylogenetic uncertainty. Sites yielding 95% *d*_N_/*d*_S_ credible intervals (CI) that do not include 1 reject neutrality and were considered to be evolving under positive selection. Following the advice of Kosakovsky Pond et al. (2005), we considered a consensus approach to identify sites evolving under positive diversifying selection. We only listed sites for which the FEL approach produces *P*-values <0.1 and for which the lower 95% CI for the RC estimate is larger than 1.

To detect diversifying positive selection in the G gene data set, we also used a random-effects likelihood (REL) [54] approach in addition to FEL and RC. This method fits a codon model to the entire alignment but allows the *d*_N_/*d*_S_ ratio to vary among sites. Positively selected sites were then identified using an empirical Bayes approach. We first tested the fit of three codon models of different complexity: a model that allows for different *d*_N_ rates among sites but keeps the *d*_S_ rate constant among sites (‘Non-synonymous’), a model that allows both *d*_N_ and *d*_S_ to vary among sites (‘Dual’), and, finally, a variant of the Dual model that also allows *d*_N_ to vary among branches in the phylogeny (‘Lineage Dual’). We used a general discrete distribution with three categories of sites to model *d*_N_ and *d*_S_ variation among sites in these substitution models. We applied the empirical Bayes method to identify positively selected sites to the best fitting codon model according to the AICM and used a log Bayes Factor larger than three as a cut-off. We only listed sites that met the cut-offs for at least two methods applied to the G gene data set and, in the results section below, listed in bold those that met the criteria for all three methods (FEL *P-*value <0.1, RC 95% lower CI value >1, REL ln BF >3).

Finally, we also investigated both pervasive an episodic diversifying selection using a recently developed mixed-effects model of evolution (MEME) [55]. MEME relaxes the assumption that the strength and direction of natural selection is constant across all lineages by allowing the distribution of *d*_N_/*d*_S_ to vary from site to site and also from branch to a branch at a site. As an extension of FEL, MEME fits a codon model with two categories of lineage-specific *d*_N_ rates to each site, one that is ≤ *d*_S_ (referred to as β^−^) and one that is unrestricted (β^+^), and tests this against a model where the unrestricted *d*_N_ is also ≤ *d*_S_ using a likelihood ratio test. We report positively selected sites with a P-value ≤0.05, which controls the rate of false positives fairly well [55], and include the estimate of β^+^ and the proportion of branches estimated to be part of this *d*_N_ rate class (*p* ^+^).

### Nucleotide Sequence Accession Numbers

The nucleotide sequences from the Dutch and Belgian RSV isolates were submitted to GenBank under the accession numbers JQ901447-JQ901458, JX015479-JX015499. Prototype reference strains A2 (M74568.1), Long (AY911262.1), Line19 (FJ614813.1) and RSS-2 (NC_001803.1) were included in protein analysis studies.

## Results

### Human RSV-A Protein Substitution Mapping

**Figure 1:**
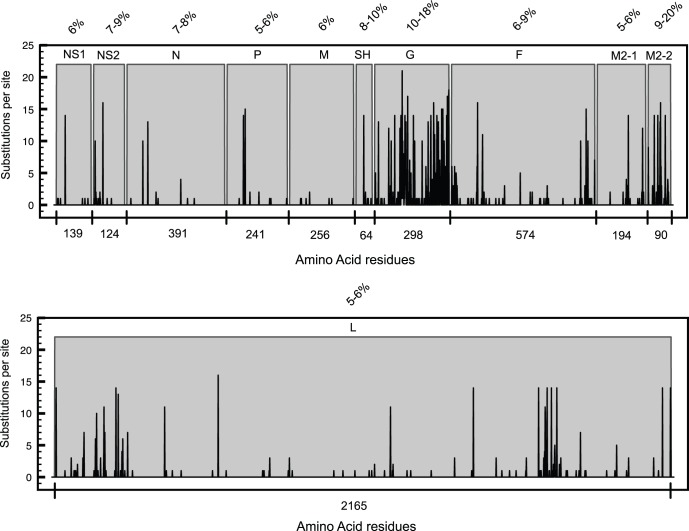
Sequence variability in RSV proteins. The number of substitutions per site (black bars) and the sequence variability (%) in each RSV protein calculated per strain relative to the consensus.

**Figure 2:**
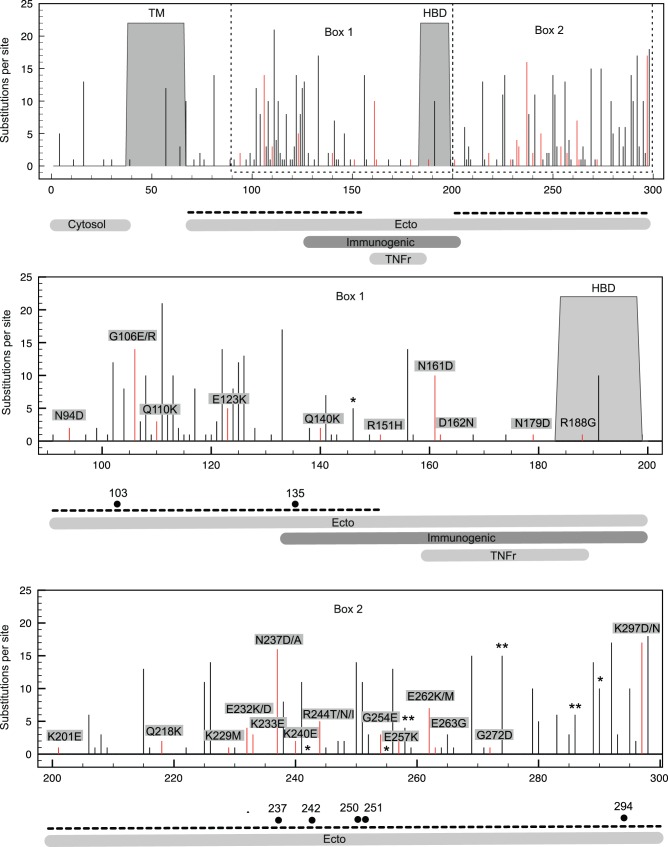
Amino acid substitutions in specific domains of the RSV G protein. Indicated are the transmembrane domain (TM), heparin binding domain (HBD), N-terminal cytosolic domain, C-terminal ectodomain, the immunogenic internal domain and the region showing partial homology with the fourth subdomain of the 55 kDa TNFr. Dashed lines represent the mucin-like regions. N-glycosylation sites are indicated by black dots, conserved substitutions by black bars and non-conserved substitutions by red bars. Positively selected sites are marked by asterisks.

Protein sequences of thirty-three Dutch-Belgian clinical strains (Table S1) and four reference lab-strains were aligned and compared to the RSV consensus. Changes in amino acid residues from all strains were mapped for each RSV protein to determine the protein substitution density for all proteins (Fig. 1). The percentage of sequence variability indicates the degree of variation from the consensus for each strain (Fig. 1 and Table S4). G and M2-2 are the most variable proteins with 10–18% and 9–20% variability, respectively. The relatively high protein sequence variation observed for RSV G suggests more relaxed selective constraints in this protein and could even indicate, as has been previously identified [56], instances of molecular adaptation. The ectodomain mucin-like regions of RSV G are two highly variable regions that are separated by a central conserved region containing the heparin-binding domain (HBD) (Fig. 2). The majority of non-conserved substitutions are located in these two regions. The C-terminal part of the RSV G immunogenic domain [57] has the highest substitution density, but non-conserved substitutions are detected throughout this domain. The RSV G immunogenic domain (a.a. 159–186), previously shown to be homologous to the TNF receptor fourth subdomain [58], shows only a few substitutions. However, the N161D substitution, as detected in both the RSV G immunogenic domain and the TNFr homologous domain, was present in ten out of thirty-seven strains. Interestingly, this residue is located in the protective epitope 152–163 of the central conserved domain of RSV G [59]. For several RSV strains, asparagine substitutions in the G protein result in the gain or loss of predicted N-glycosylation sites either by direct changes in the Asn-Xaa-Ser/Thr N-glycosylation sequons or by alterations in the surrounding sequence (Table S5).

**Figure 3:**
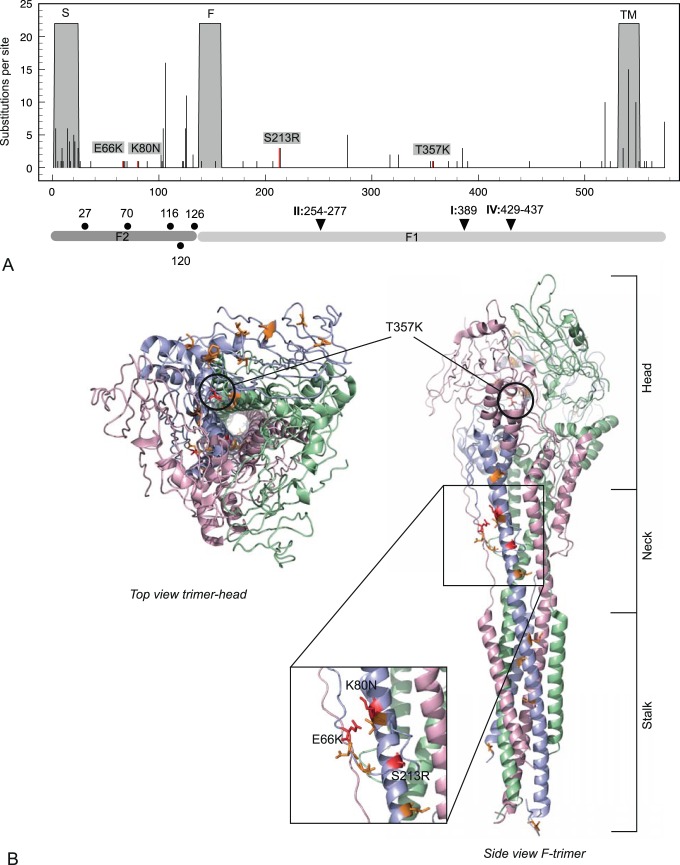
Amino acid substitutions in specific domains of the RSV F protein. A) Indicated are the signal peptide (S), fusion peptide (F), transmembrane domain (TM), F1 and F2 domains, antigenic sites I, II and IV (black triangles), and N-glycosylation sites (black dots). B) Substitutions marked in the 3D structure of the trimeric fusion protein. Amino acid class changes are indicated in red, orange indicating similar class.

The RSV F signal peptide that is involved in F protein translocation to the ER [60] is a major substitution hotspot (Fig. 3A) whereas other parts of RSV F are rather conserved. Most substitutions in the mature F protein are located at the surface of this fusion protein (Fig. 3B), which, therefore, might affect either antibody binding or interactions with cellular receptors. However, previously recognized antigenic regions [61] appear to be highly conserved. Non-conserved substitutions in the fusion protein are only found in the prototype strains RSV-Long (K80N, S213R) and RSV-line19 (E66K, S213R, T357K), as well as in the clinical strain 05-000417 (S213R). The E66K and K80N replacements are located in the F2 domain, which is involved in direct interaction with host cells and, accordingly, is a determinant of host species specificity. N-glycosylation sites are predicted for amino acid residues 27, 70, 116, 120 and 126. All of these are situated within the F2 domain and, in contrast to the N-glycosylation sites predicted for RSV G, are highly conserved with the exception of reference strain RSV-A2 and clinical strain 01-002279 that did not show N-glycosylation potential at position 120. The site at position 500 which was earlier predicted to have glycosylation potential [62] was not identified as such here (glycosylation potential <0.5).

### Potentially Artifactual Recombination Signals within Some RSV Genomes

An initial screen for recombination within a dataset containing the 39 RSV sequences available from the study of Kumaria *et al.* (2011) and Rebuffo-Scheer *et al.* (2011), together with the 33 newly determined sequences described here, revealed evidence of approximately 64 unique recombination events within the evolutionary histories of these sequences. Collectively 55 of these recombination events were clustered within the 14 RSV genome sequences deposited by Kumaria *et al*. (2011). The pattern of recombination within these sequences was extremely complex with, for example isolate RSV-12 (accession number GU591769) containing at least 10 recombination breakpoints and genome regions apparently derived from at least five different parental viruses.

The fact that signals of recombination were mostly concentrated within a set of genomes all determined within a single research laboratory suggested that these genomes might all be recombinant sequence assembly or amplification artifacts. Using the permutation-based recombination breakpoint-clustering test employed in [63], we detected an extremely significant association (p<0.0001) between breakpoint positions and the locations of the primer binding sites used to sequence these genomes. This very strongly suggests that the recombination signals detectable within these sequences are sequence assembly artifacts and we therefore excluded these sequences from subsequent recombination analyses.

Further recombination analyses indicated that five of the Milwaukee isolates, A/WI/629-4302/98, A/WI/629-4285/98, A/WI/629-3734/98, A/WI/629-4239/98 and A/WI/629-4111/98 [36] were detectably recombinant (collectively containing evidence of six recombination events; Figure S1). As the majority of these recombinant genomes (>70% of their sequences) were apparently derived from a single major parental virus, they were included in subsequent analyses with, in a “removed” dataset, the fragments derived from the minor parental viruses removed or, in a “distributed” dataset, the fragments derived from the different parents being separated into two different sequences.

Seven out of eleven Dutch-Belgian strains that were sequenced via the 454-sequencing method also showed some degree of recombination. After re-analyzing these (Table S1) strains by conventional Sanger sequencing, recombination events were no longer present confirming that short-read sequencing techniques and their associated contig-assembly methods can, in many cases produce artifactual recombinant viral genomes: A fact that should encourage all future studies of RSV whole genome sequences to explicitly account for recombination.

### A Bayesian Reconstruction of RSV-A Complete Genome Evolutionary History

**Figure 4:**
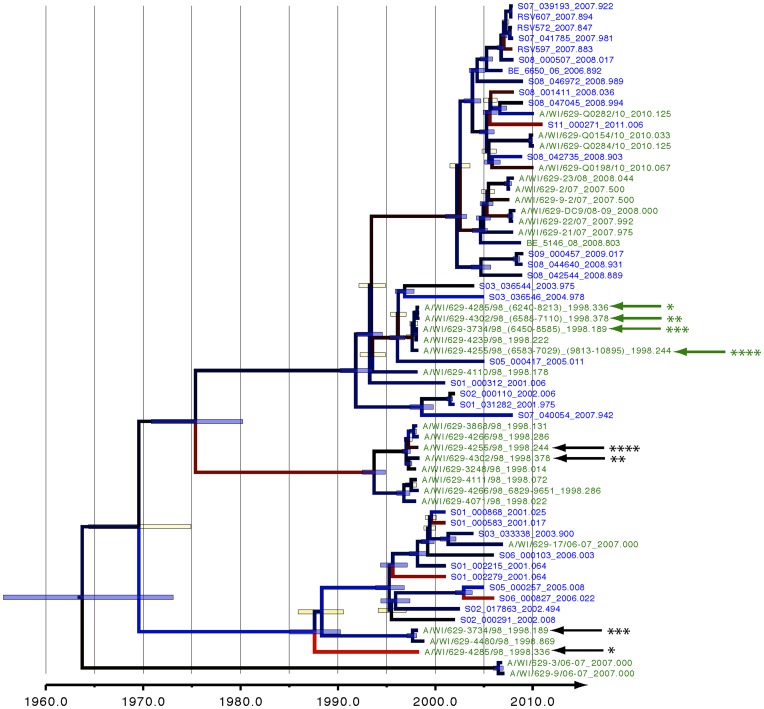
Phylogeny based on whole genomic sequences. Distribution of Dutch-Belgian strains (blue) and Milwaukee strains (green). A color gradient (blue = slow, black = average, red = fast) reflects the variation in evolutionary rates among branches; node bars depict the credibility intervals for nodes showing a posterior probability support >95% (blue bar) or <95% (yellow bar). Recombinant partitions are indicated with arrows and asterisks.

An exploratory linear regression analysis of root-tip-divergence as a function of sampling time indicated that there is a clear temporal signal for nucleotide divergence in the RSV-A complete genomes sampled over the last thirteen years (Fig. S2), justifying the application of a dated tip molecular clock model in the Bayesian evolutionary reconstruction. We compared a strict and relaxed clock model using different approaches to assess model fit. Although this did not provide consistent results (Table S6), path sampling and stepping-stone sampling, which have recently been shown to provide the most accurate results [47], both rejected the assumption of a constant substitution rate across phylogenetic tree branches and hence we used this model in further analyses. We represent the posterior tree distribution for the ‘distributed’ RSV-A complete genome data set using a maximum clade credibility (MCC) tree in Figure 4. The corresponding parts of each mosaic genome identified using RDP3 do not cluster together in this tree (green and black arrows/stars in the figure), which confirms that these genome regions are likely derived from genetically divergent viruses. The smaller genomic stretches extracted from these mosaic genomes are generally very closely related to other Milwaukee genomes, with four of them nearly identical to one another, highlighting the nonrandom nature of these mosaic patterns. The MCC tree for the ‘removed’ RSV-A complete genome data set had the same topology as for the ‘distributed’ RSV-A data set (data not shown).

The complete genome phylogeny does not indicate geographical or temporal clustering patterns because the Dutch-Belgian and Milwaukee strains are phylogenetically interspersed and strains from different epidemic seasons are present in the same clusters. For further classification of our new strains, we compared the G gene sequences extracted from the complete genomes to previously described sequences that have been assigned to specific subgroup A genotype lineages GA1, GA2, GA4, GA5 or GA7 [36], [64] (Fig. S3). The Dutch-Belgian strains predominantly belong to the GA2 and GA5 lineages, and in this study, six Milwaukee strains (A/WI/629-4071/98, A/WI/629-3248/98, A/WI/629-4302/98, A/WI/629-4255/98, A/WI/629-3868/98 and A/WI/629-4266/98), which were previously assigned to the GA7 lineage, were classified as genotype GA2 in our analysis.

**Figure 5:**
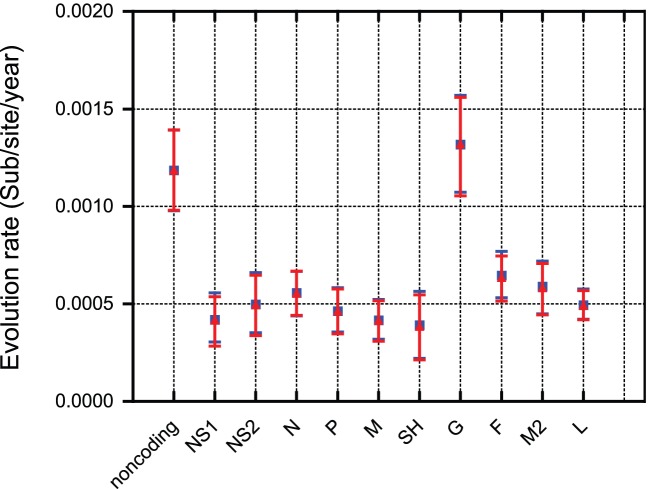
Evolutionary rate estimates for the different genes and for the non-coding partition. Distributed (blue square) and removed (red triangle) data sets.

The RSV-A complete genome evolutionary history dates back to 1964 (95% credibility interval between 1956 and 1973). The inclusion of older strains sampled in 1954 and 1961 in the G gene pushes the MRCA further back in time (1943 [1923,1954], Table 1). The Bayesian skyline model indicated that RSV populations over the past 70 years have been characterized by a constant degree of relative genetic diversity through time (for both the distributed and the removed data sets, Fig. S4). The genomic evolutionary rate was estimated to be 6.47×10^−4^ substitutions per site per year (95% credibility intervals ranging from 5.56×10^−4^ to 7.38×10^−4^), which is considerably slower than rates previously estimated using only the G gene (1.83×10^−3^; [64]). Therefore, we investigated evolutionary rate variability across the genome by partitioning the various genes and the non-coding regions in our Bayesian estimation approach (Fig. 5). This revealed elevated substitution rates in the non-coding regions and the G gene. The latter also has the highest sequence variability score (5–9% variability; Fig. S5). The relatively high rate we have estimated for the G gene partition is in good agreement with previous estimates of substitution rates in this gene [64] and indicates relaxed selective pressures operating on the G gene relative to other RSV genes. Specifically, other genes were considerably more conserved than the G gene and showed substitution rates that were 3- to 4-fold lower than those of the G gene.

**Table 1:** Evolutionary rate and TMRCA estimates.

			95% HPD	
		Mean	Lower	Upper
Mean rate(substitutions/site/year)	Distributed	6.47E−04	5.56E−04	7.38E−04
	Removed	6.44E−04	5.52E−04	7.46E−04
	G gene	2.22E−03	1.93E−03	2.56E−03
TMRCA (date)	Distributed	1964	1957	1972
	Removed	1964	1956	1973
	G gene	1942	1929	1953

### Site-specific Patterns of Selective Pressure

To identify positively selected sites in the RSV-A genome, we used a fixed-effects likelihood (FEL) approach and a recently developed renaissance counting (RC) method to evaluate nonsynomous/synonymous substitution rate ratios (*d*_N_/*d*_S_) at each codon position. Table 2 lists sites that are apparently evolving under positive selection given the consensus cut-off values we propose for both methods (see Methods). The sites marked in bold also meet more stringent cut-offs for one of the two methods. As expected from the evolutionary rate variability across the genome, the majority of positively selected sites are found in the G gene, which can be interpreted as evidence for the action of diversifying selection at these sites. Single positively selected sites were detected in the L gene (site 104), the N gene (site 216) and the M2 gene (site 117; with support for the latter two sites being lower than that for the L gene site).

**Table 2:** Site-specific positive selection pressure.

			RC	FEL		REL	
Data set	Positive selected site	Gene	dN/dS	dN-dS	P value	E[dN/dS]	Log(BF)
Full genome	**286**	G	2.62 (2.06,3.95)	9.49	<0.01	N/A	N/A
	**244**	G	2.54 (2.04,3.72)	8.12	0.1	N/A	N/A
	**274**	G	2.51 (2.05,3.69)	6.43	0.02	N/A	N/A
	**250**	G	2.11 (1.71,3.09)	5.17	0.01	N/A	N/A
	**104**	L	1.73 (1.37,2.65)	3.55	0.08	N/A	N/A
	**101**	G	1.72 (1.29,2.59)	3.67	0.04	N/A	N/A
	**258**	G	1.72 (1.38,2.51)	3.86	0.04	N/A	N/A
	216	N	1.32 (1.05,1.99)	4.15	0.1	N/A	N/A
	117	M2	1.30 (1.00,1.99)	4.31	0.1	N/A	N/A
	**237**	G	1.29 (1.05,1.89)	4	0.04	N/A	N/A
G gene	**274**	G	4.08 (2.63,6.39)	3.11	0.01	2.73	4.3
	**290**	G	3.19 (2.04,4.95)	1.81	0.03	2.71	4.14
	244	G	2.81 (1.83,4.34)	1.99	0.11	2.48	3.26
	**258**	G	2.52 (1.65,3.94)	1.58	0.03	2.68	3.99
	**146**	G	2.19 (1.33,3.38)	1.31	0.04	2.47	3.18
	**286**	G	2.00 (1.32,3.16)	1.67	0.05	2.7	4.13
	285	G	1.92 (1.25,2.98)	1.05	0.08	1.65	1.37
	**242**	G	1.91 (1.24,2.99)	1.92	0.02	2.55	3.45
	**255**	G	1.90 (1.25,2.92)	1.72	<0.01	2.55	3.41
	132	G	1.16 (0.74,1.82)	2.12	0.03	2.71	4.15

Sites marked in bold are positive selected sites that meet the criteria of all applied methods. Underlined sites are positive selected sites that agree with the G gene and full genome analysis. RC = Renaissance Counting, FEL = Fixed-Effect Likelihood, REL = Random-Effects Likelihood, dN = non-synonymous substitution, dS = synonymous substitution and log(BF) = Bayes Factor logarithm. REL could not be acquired for the whole genome data set (N/A).

To further scrutinize the instances of diversifying selection in the G gene, we analyzed the larger G gene data sets with the same methods and an additional random-effects likelihood (REL) approach. For the latter, we first tested, using an AIC-based approach, whether the applied codon substitution model needed to account for nonsynonymous and synonymous rate variability across sites and/or branches (Table 3). We found strong evidence for both nonsynonymous and synonymous rate variability across sites, but no support for a model that also accommodates a separate nonsynonymous rate for each branch. Sites that were found positively selected according to the cut-offs for at least two methods are listed in Table 2; sites that meet the criteria for all three methods are listed in bold. There is only partial agreement between the sites identified in the G gene data (underlined in Table 2) and those found to be positively selected based on the G genes sampled from the complete genome dataset. Most positively selected sites were detected within the two hyper-variable regions of the G protein ectodomain (marked with an * in Figure 2). The positively selected site 242 is a predicted N-glycosylation site and substitutions can result in loss of glycosylation.

**Table 3:** Model-fit of random-effect likelihood (REL) models.

Model	Log L	AIC	cv(dS)
Nonsynonymous	−6376.14	13506.28	0
Dual	−6345.39	13452.78	7.29
Lineage Dual	−6181.06	13856.12	13.18

Log L = logarithm of the maximum lilelihood value for the model.

AIC = value of the Akaike Information Criterion model selection index.

CV(dS) = coefficient of variation of the synonymous substitution distribution.

The methods we applied to investigate site-specific selective patterns will generally identify sites that are under positive diversifying selection in most lineages of the tree (pervasive diversifying selection). However, sites may also experience diversifying selection in a restricted number of branches (episodic diversifying selection). To discriminate between these processes, and to identify sites under episodic diversifying selection in particular, we applied a recently developed approach that relaxes the assumption of constant diversifying selection throughout time (MEME, [55]; Table 4). MEME identified 16 sites under positive diversifying selection (P-value ≤0.05), six of which were already pinpointed by the other methods (in bold in Table 4). Not surprisingly, there is a large proportion branches estimated to be under diversifying selection (*p^+^*) for these six sites. Most other sites are candidates for sites under episodic diversifying selection, some of which only implicate a small subset of branches but with high nonsynonymous substitution rates (β^+^). To contrast the substitution history for sites under pervasive and episodic selection, we mapped the amino acid residues for four G gene sites to the internal MCC tree nodes in Figure S6. This demonstrates that the amino acid patterns for the two sites with the highest RC *d*_N_/*d*_S_ estimates (site 274 and 290 under pervasive selection), are the result of a relatively rich substitution history on both external an internal branches, whereas substitutions for the two sites under episodic selection (site 154 and 255) occur on only three external branches and one external branch respectively. We caution against over-interpreting the latter because a single substitution on an external branch may also reflect a deleterious mutation that has been weeded out by purifying selection [65].

**Table 4:** Sites under episodic diversifying selection in the G gene data set.

Positive selectedsite	β^+^	*p^+^*	P value
154	2010.12	0.01	0.00
255	19.06	0.09	0.00
**274**	3.48	1.00	0.00
224	113.89	0.01	0.01
**290**	2.12	1.00	0.02
256	23.86	0.13	0.02
297	18.83	0.11	0.03
250	21.85	0.12	0.03
**286**	2.47	1.00	0.03
**132**	1.67	1.00	0.03
**146**	1.50	1.00	0.04
247	1.18	1.00	0.04
273	1.37	0.64	0.04
218	252.07	0.01	0.04
**258**	3.95	0.44	0.05
124	3.96	0.44	0.05

Sites marked in bold are positive selected sites that meet the criteria of all applied methods.

### The Neutral Phylodynamics of RSV-A

To test the impact of diversifying selection in the G gene on virus circulation patterns, we conducted a genealogical test of neutrality using posterior predictive simulation. Comparison of the tree shapes inferred from the complete genomes to those obtained by simulating under the neutral model with the same population dynamics indicated that neutrality could not be rejected at the population level (P = 0.24). The same holds true for the G gene data set although the inclusion of older samples and the resulting longer time scale may have resulted in a lower *P*-value in this case (P = 0.08).

## Discussion

Here we present the first study describing RSV-A whole-genomic diversity through time using phylodynamic inference. We mapped the rate and density of nucleotide substitutions in viral gene products to provide an indication of the relative importance of individual protein evolutionary dynamics in RSV replication or immune evasion. The evolutionary and population dynamics of RSV-A were estimated using a Bayesian coalescent approach, based on two different datasets of sequences from time-stamped strains collected between 2001 and 2011. One set was recently collected in the Milwaukee area [36]. Like previous molecular evolution studies [64], [66], Rebuffo-Scheer *et al* [35], [36] described the epidemiology of these strains according to G gene genotyping. The current study adds another set of thirty-three complete genomic sequences to the literature, enabling in-depth examination of RSV evolutionary dynamics by Bayesian genealogical inference. Although the collective sampling arose from a few specific locations, the general absence of population substructure and the co-circulation of different lineages through time ensure that we analyzed a representative RSV-A genomic sample.

Most nucleotide sequence variability was detected in the non-coding regions, as well as the M2-2 and G genes. The role of intergenic non-coding regions and their high sequence variability is unknown. In the G and M2-2 proteins high nucleotide sequence variability is correlated with elevated amino acid substitution rates. Variability in the M2-2 transcription regulatory protein sequences could largely be attributed to substitutions in reference strains (Long, Line19, and A2), suggesting that this variability may be attributable to adaptation to artificial growth conditions during long-term culturing. Given the role of M2-2 in switching from transcription to RNA replication during the virus infection cycle, together with our observation that none of the clinical strains shows such increased variability, we hypothesize that the absence of immunological constraints on the cultured isolates may have allowed changes in this switching protein that facilitate more efficient packaging and increased viral yields *in vitro*. We did not detect any difference in the nucleotide sequence of three RSV strains sequenced either directly from patient isolates or after passaging on HEp2 cells for three culture rounds (data not shown). Apparently, short-term culturing does not result in a noticeable accumulation of culture adaptive substitutions and it may therefore be acceptable to use whole genomic sequences from RSV isolates with low culture passage levels in future genetic analyses. The G protein is the virus’ most immunogenic protein and it has been suggested that neutralizing antibodies drive positive selection for immune escape variants of the ectodomain [67]. All other RSV genes show high degrees of conservation, suggesting that the impact of immune pressures on the evolution of these genes is minimal. Nevertheless the substitutions observed in these genes might very well affect RSV fitness and its transmission dynamics. The exact mapping of conserved sites throughout the genome might be very useful for both future research into the biological functions of these regions as well as for the development of therapeutic and preventive strategies.

An important prerequisite to performing phylogenetic inference is a thorough check of recombination events occurring in the genomes being analyzed. Recombination in RSV genomes is unprecedented in natural isolates and has only very rarely been detected under controlled laboratory conditions [68]. Therefore, it is not very likely that any of the recombination events detected here are the product of natural RSV evolutionary processes. Indeed, our analyses suggest that in both the case of sequences deposited by Kumaria *et al*. (2011), as well as in seven of our own 454-derived sequences, the recombinant sequences are likely to be either PCR or sequence assembly artifacts. Especially our own findings underscore the potential pitfalls of applying phylogenetics-based analytical approaches to sequences derived through the application of short-read sequencing techniques. To incorporate published strains with recombination signal, we compared a data set where minor recombinant parts were removed and a data set where sequence parts with different phylogenetic clustering were distributed over multiple taxa. These data sets yielded highly similar evolutionary rate and demographic estimates, but we noted that a large number of such distributed strains may complicate the interpretation of demographic inferences. This is because the relationship between viral coalescent rates and the change in effective number of infections through time only holds when each individual is represented by a single sequence.

To assess whether the genotype grouping is consistent when different genomic regions are used, we compared the phylogenetic trees constructed from all single genes, as well as the whole genome based tree. The analysis of the non-recombinant complete genome strains revealed consistent genotype clustering patterns constructed from all single genes, including the most variable G gene (data not shown). Therefore, we conclude that genotyping based only on G gene sequences, appears to be a suitable method for assigning genotypes to RSV strains. However, in general there are no equidistant clusters corresponding to RSV genotypes and differences in the analyzed strain population, i.e. typing strains against different background populations, might give rise to different classifications as observed for six Milwaukee strains (A/WI/629-4071/98, A/WI/629-3248/98, A/WI/629-4302/98, A/WI/629-4255/98, A/WI/629-3868/98 and A/WI/629-4266/98), which were previously assigned to the GA7 lineage, but are classified as genotype GA2 in our analysis. This indicates that RSV genotyping may be intrinsically volatile.

Changes in amino acid sites that are either N-glycosylated or susceptible to selective pressure correlate with RSV phenotypic differences and variations in RSV infectiousness. This may ultimately affect the outcome of RSV infection and transmission dynamics. We studied the N-glycosylation sites within the two major RSV glycoproteins, F and G. Here we observed differences in the predicted N-glycosylation sites for the F protein when comparing our data to those of Zimmer *et al* [62]. Modifications in asparagine residues and the related alterations in N-glycosylation potential could affect RSV G and F protein folding, possibly resulting in altered virus-host attachment, changes in immune evasion strategies or distorted fusion abilities. Zimmer *et al* used the RSV-Long strain to conduct radio-immunoprecipitations with recombinant RSV F mutants to determine the N-glycan positions. They reported glycosylation at N27, N70 and N500. Here, position N500, which is known to be required for efficient syncytia formation, was not predicted to be glycosylated, underscoring the need for *in vitro* confirmation of predicted N-glycosylation sites. In addition, N-glycosylation is not only determined by sequence composition, but also heavily depends on the type of host cells that are infected [69].

Selective pressure analysis showed that the RSV genes have predominantly negatively selected, or neutrally evolving sites. Only the RSV G gene displays clear evidence of pervasive positive diversifying selection. Using the full genome dataset we detected positively selected sites at G protein positions 101, 237, 244, 250, 258, 274, and 286. In the larger G gene exclusive dataset, additional positively selected sites at positions 146, 242, 255 and 258 were detected by the various detection methods (Table 2). Positively selected site 237 has an asparagine residue with N-glycosylation potential [18] for approximately 50% of the strains included in this study. More positively selected sites were found at predicted N-glycosylation positions 242, 244, 250 and 258. Substitutions at positions 274 and 286 have previously been associated with antibody escape [70]. Less is known about the effects of amino acid replacements at sites 101, 146, and 255, although positions 101 and 146 fall within the immunogenic region of RSV G [57]. Clearly, the exact G gene sites identified as evolving under positive selection are not consistent among different studies [64], [71]. The vast majority of previously described sites [59], [72] were not predicted here, even when using the G gene only data set. Among the sites predicted in this study, positions 242, 255 and 258 were also predicted by Botosso *et al.* (2009) [71]. Differences in positively selected sites can be attributed to different data sets and/or the use of different analytical methods. For example, unaccounted recombination is known to yield false evidence of positive selection. The fact that the amount of sequence data strongly impacts the power of selection analyses is evident from the positively selected sites in the G gene when comparing the smaller complete genome data set to the larger G gene specific data set. Our codon model comparison also indicates an important degree of variation in synonymous substitution rates among sites, which could result in sites being falsely identified as evolving under positive diversifying selection if not accounted for. The sensitivity to sequence sampling and the amount of data is less of an issue for the recently developed MEME approach [55], which also captures episodic diversifying selection by disentangling lineages under purifying and positive selection for each site. MEME confirmed six sites under pervasive diversifying selection, but also identifies a considerable number of sites under episodic diversifying selection. This confirms the notion that site-specific positive selection may have been largely underestimated because purifying selection in many lineages can mask episodic adaptation.

The degree of phylogenetic intermixing of samples from different geographical regions in the maximum clade credibility tree indicates that there is likely very little, if any, temporal or long-term geographical structuring of RSV populations. Several genotypes may co-circulate and selection leading to the replacement of predominating genotypes is likely to be far more sporadic than that which, for example, occurs with influenza viruses where high host population-wide prevalence’s of antibodies targeting the hemagglutunin (HA) and neuraminidase (NA) genes are able to cause extinction of specific clades. In contrast to human Influenza A [50] and Norovirus GII.4 [51], no evidence of selection-driven viral population turnover was uncovered by our posterior predictive analysis of the whole genome (P = 0.24), which indicates that substitutions are generally random. This, in combination with the high degree of variability detected for the G protein allows us to conclude that the RSV attachment process, mediated by N- and O-linked sugars of the RSV G attachment glycoprotein, does not put significant evolutionary constraints on the selection of circulating variants. Consequently, the RSV G protein is not expected to induce functional B-cell mediated immunity and is therefore unlikely to be an effective vaccine target. For the RSV F protein the situation is completely different given the fact that antibodies targeting RSV F (e.g. RespiGam®, Synagis®) are clearly protective when administered as RSV prophylactics. Our inability to detect RSV F escape mutants indicates that immune evasion mutations in this gene are either rare, episodic, or have a strongly detrimental effect on virus viability. This reinforces the suitability of RSV F as an effective vaccine target.

In conclusion, our evolutionary analysis indicates that the RSV genome is largely conserved and, with the exception of the G protein, almost exclusively accumulates neutral substitutions. There is very little evidence to suggest that the evasion of adaptive host immunity is a primary driver of the observed RSV diversity. This might reflect a situation in which individuals with immune systems that attempt to acquire immunologic memory of the virus destabilize the immunologic balance of infections, both decreasing their tolerance for RSV and, increasing the likelihood that they will experience an enhanced disease outcome. Based on these findings we suggest firstly that future control strategies should target the RSV F protein and, secondly, that these strategies might be potentiated by co-therapeutic strategies modulating natural host immune responses.

## Supporting Information

Figure S1 **Evidence of recombination events within the genomes of RSV isolates.** (A) A/WI/629-4266/98; (B) A/WI/629-4302/98; (C) A/WI/629-4285/98; (D) A/WI/629-3734/98; and (E) A/WI/629-4255/98. Whereas bootscan plots indicate regions of sequence where these isolates are differentially most closely related to major (in green) and minor (in blue) parental viruses, peaks in the Maxchi plots indicate the most probable locations of recombination breakpoints. Neighbor joining trees (Jukes Cantor model with 100 bootstrap replicates) constructed from different regions of the RSV full genome alignment (where region A corresponds with the genome region inherited from the recombinant’s major parent and regions B and C correspond with the genome regions inherited from the recombinant’s minor parent/parents). Within these trees, recombinant sequences are highlighted in red, sequences closely related to the recombinants’ major parents are highlighted in green, and sequences closely related to the recombinants’ minor parents are highlighted in blue.
(ZIP)

Figure S2 **Root-to-tip divergence plot.** A) Distributed data set, B) Removed data set
(TIF)

Figure S3 **Phylogeny of the RSV G gene.** The accession numbers, country of isolation, isolation date and references for the sequences included are listed in Table S3.
(TIF)

Figure S4 **Bayesian Skyline plot reconstructions depicting the superimposed estimated change in effective population size through time for the distributed (blue) and removed (red) data sets.**
(TIFF)

Figure S5 **Mutational hotspots and nucleotide sequence variability within the RSV genome.** The number of substitutions per site (black bars) and the nucleotide sequence variability (%) in each RSV gene calculated per strain relative to the consensus.
(TIFF)

Figure S6 **Pervasive and episodic selection sites in the G gene.** The G gene based phylogenetic trees show the substitution history for episodic sites 154 and 255 plus the pervasive sites 274 and 290.
(ZIP)

Table S1 **Patient and sample information.**
(DOC)

Table S2 **Primers used for cDNA preparation and whole genome sequencing.**
(DOC)

Table S3 **Genbank human RSV-A sequences used in this study.**
(DOC)

Table S4 **Overview protein sequence variability per RSV strain.**
(DOC)

Table S5 **N-glycosylation probability report of Asn-Xaa-Ser/Thr sequons in the RSV G protein.**
(DOC)

Table S6 **Clock comparison for marginal likelihood estimates.**
(DOC)
