# Supplementary figures and images for: Genetic Variability among Complete Human Respiratory Syncytial Virus Subgroup A Genomes: Bridging Molecular Evolutionary Dynamics and Epidemiology
Source: PLoS One. 2012 Dec 7;7(12):e51439. doi: 10.1371/journal.pone.0051439 (PMC3517519; doi:10.1371/journal.pone.0051439)

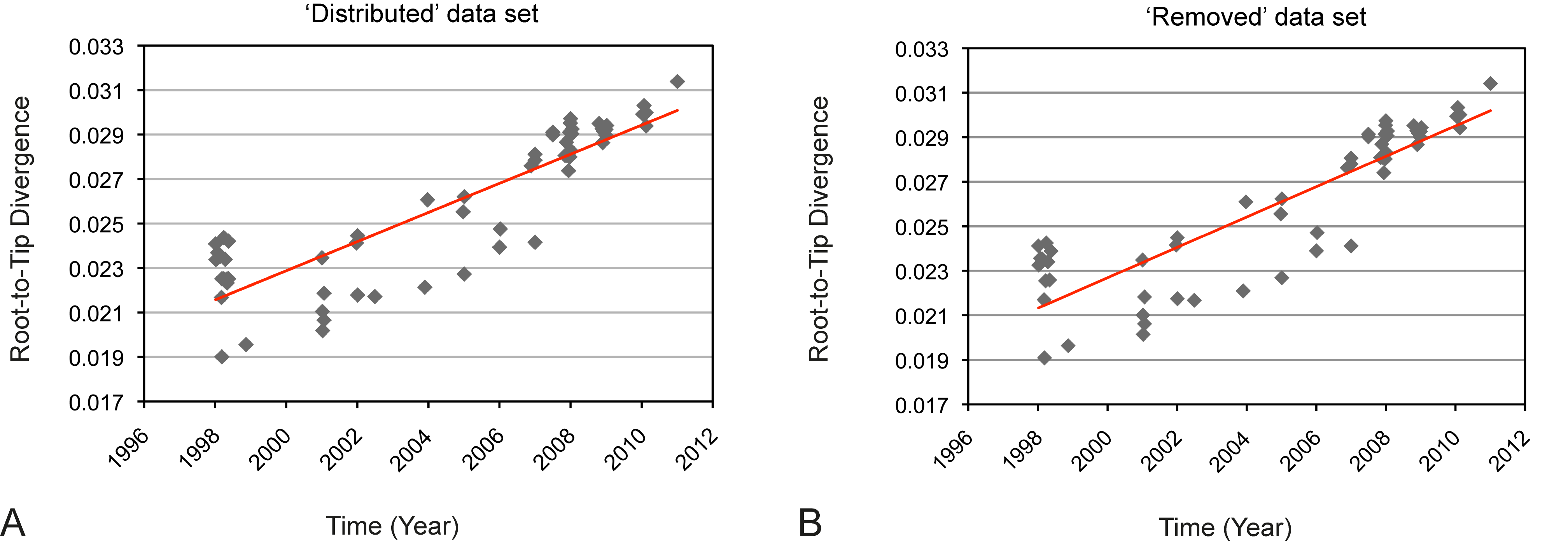

Supplement: Figure S2 — Root-to-tip divergence plot. A) Distributed data set, B) Removed data set (TIF) [file pone.0051439.s002.tif]

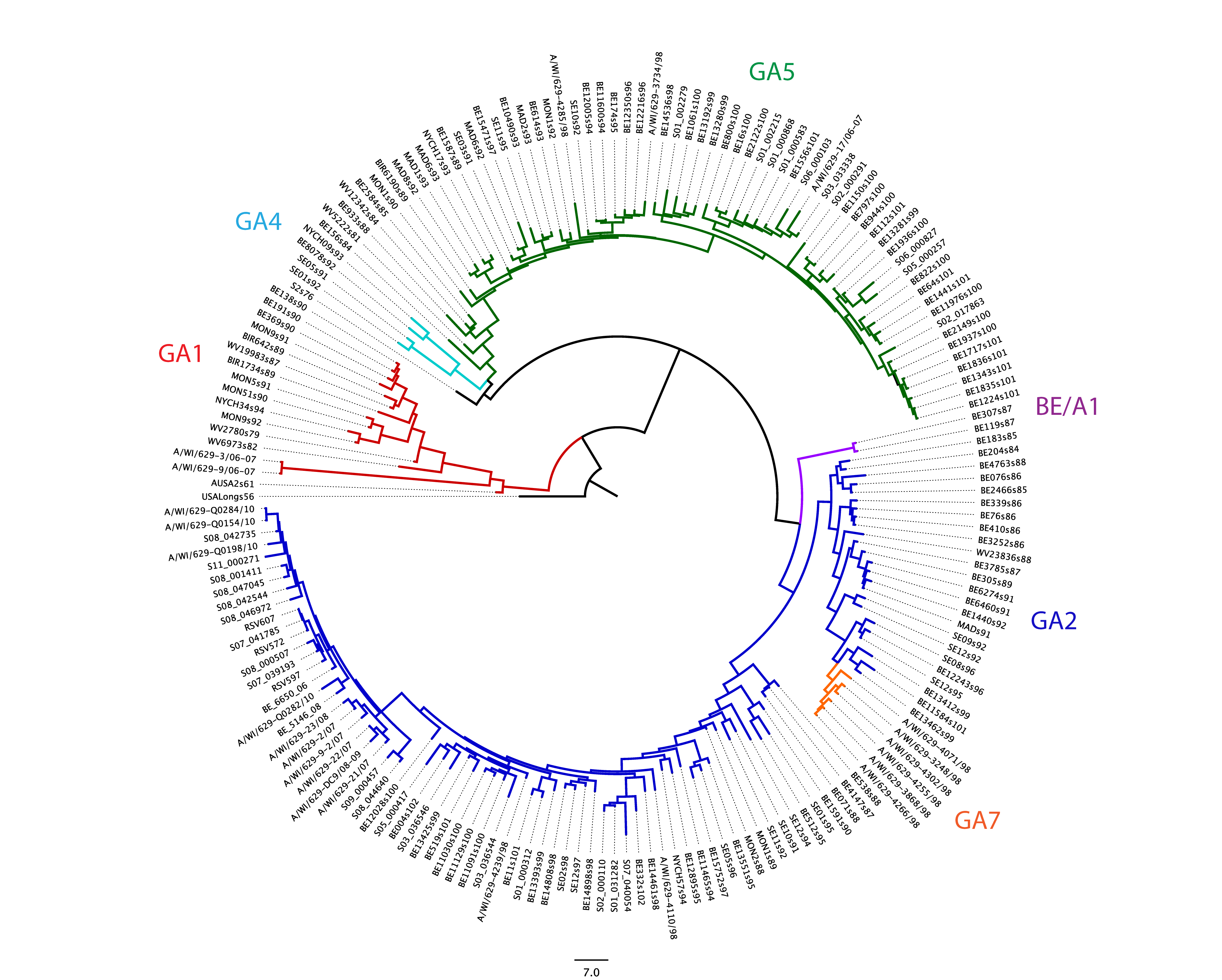

Supplement: Figure S3 — Phylogeny of the RSV G gene. The accession numbers, country of isolation, isolation date and references for the sequences included are listed in Table S3. (TIF) [file pone.0051439.s003.tif]

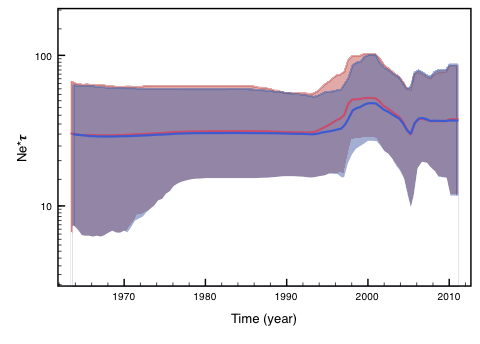

Supplement: Figure S4 — Bayesian Skyline plot reconstructions depicting the superimposed estimated change in effective population size through time for the distributed (blue) and removed (red) data sets. (TIFF) [file pone.0051439.s004.tiff]

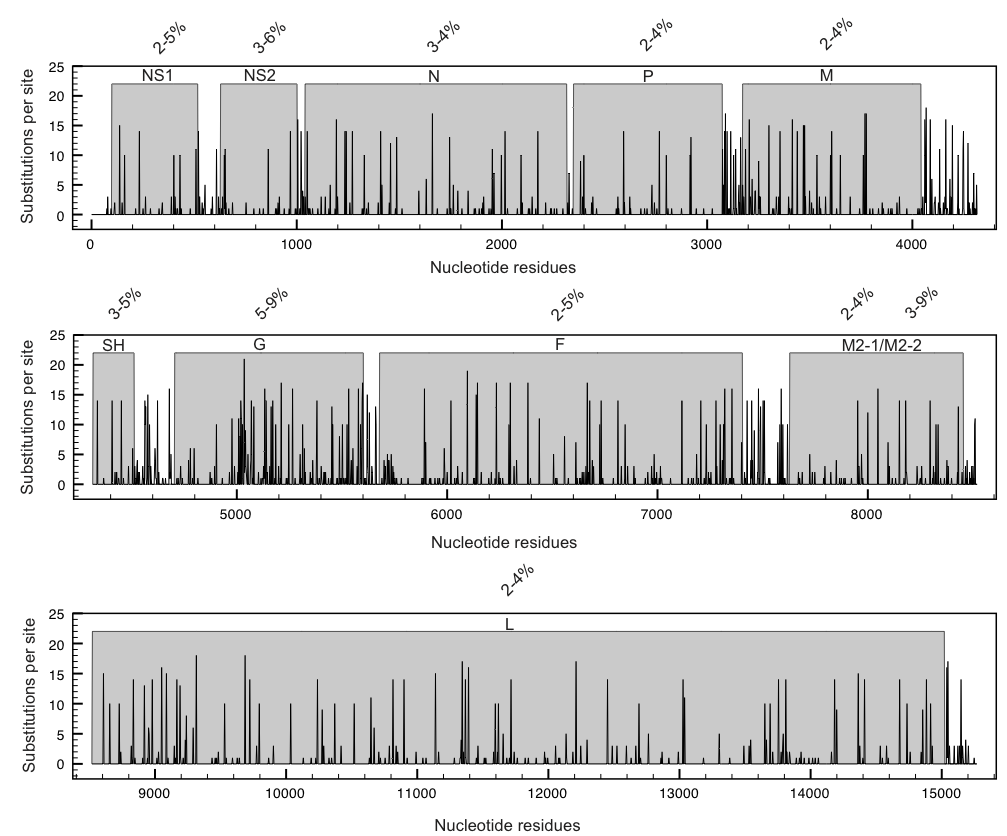

Supplement: Figure S5 — Mutational hotspots and nucleotide sequence variability within the RSV genome. The number of substitutions per site (black bars) and the nucleotide sequence variability (%) in each RSV gene calculated per strain relative to the consensus. (TIFF) [file pone.0051439.s005.tiff]

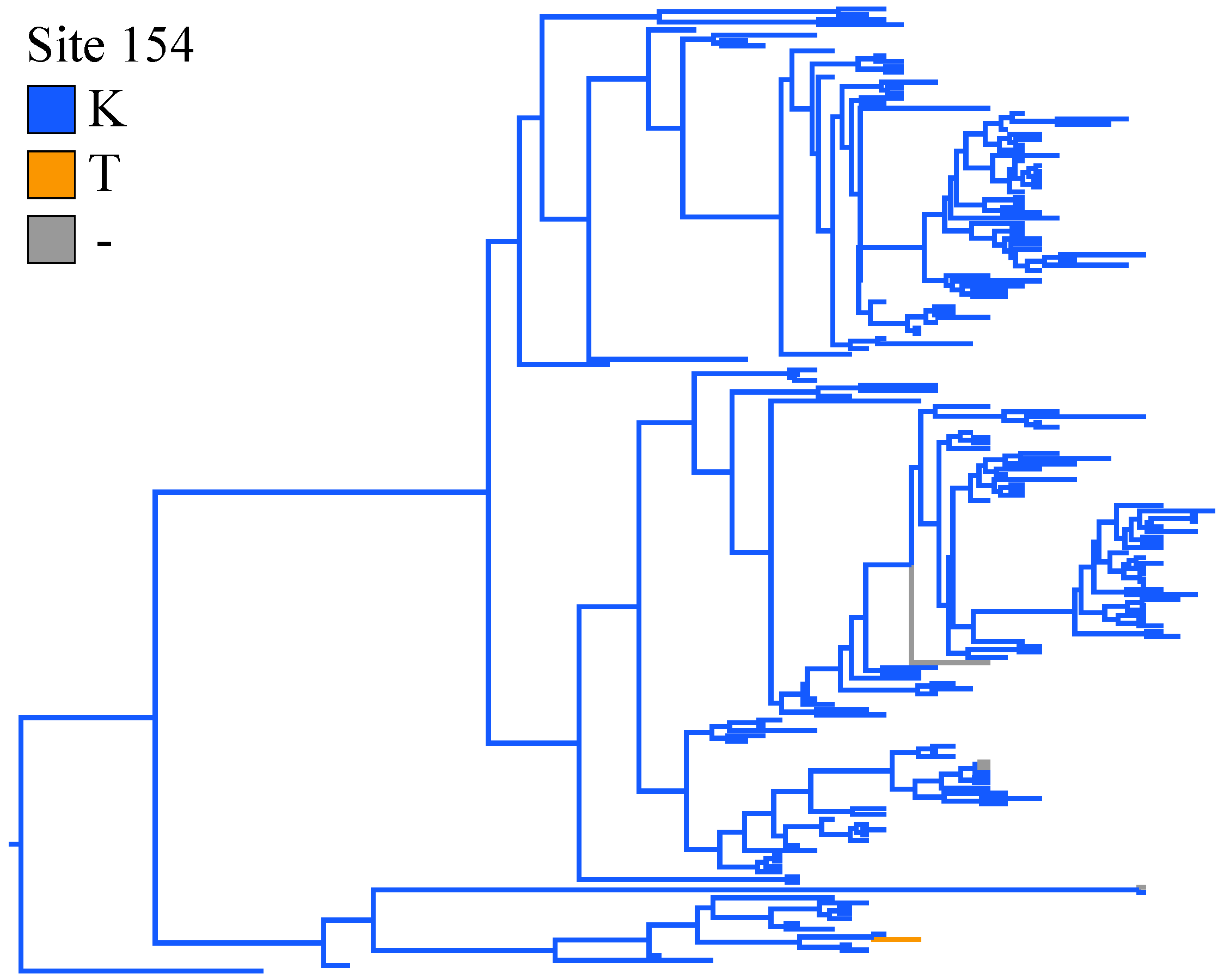

Supplement: Figure S6 — Pervasive and episodic selection sites in the G gene. The G gene based phylogenetic trees show the substitution history for episodic sites 154 and 255 plus the pervasive sites 274 and 290. (ZIP) [file pone.0051439.s006.zip › Figure S6 site 154.tiff]

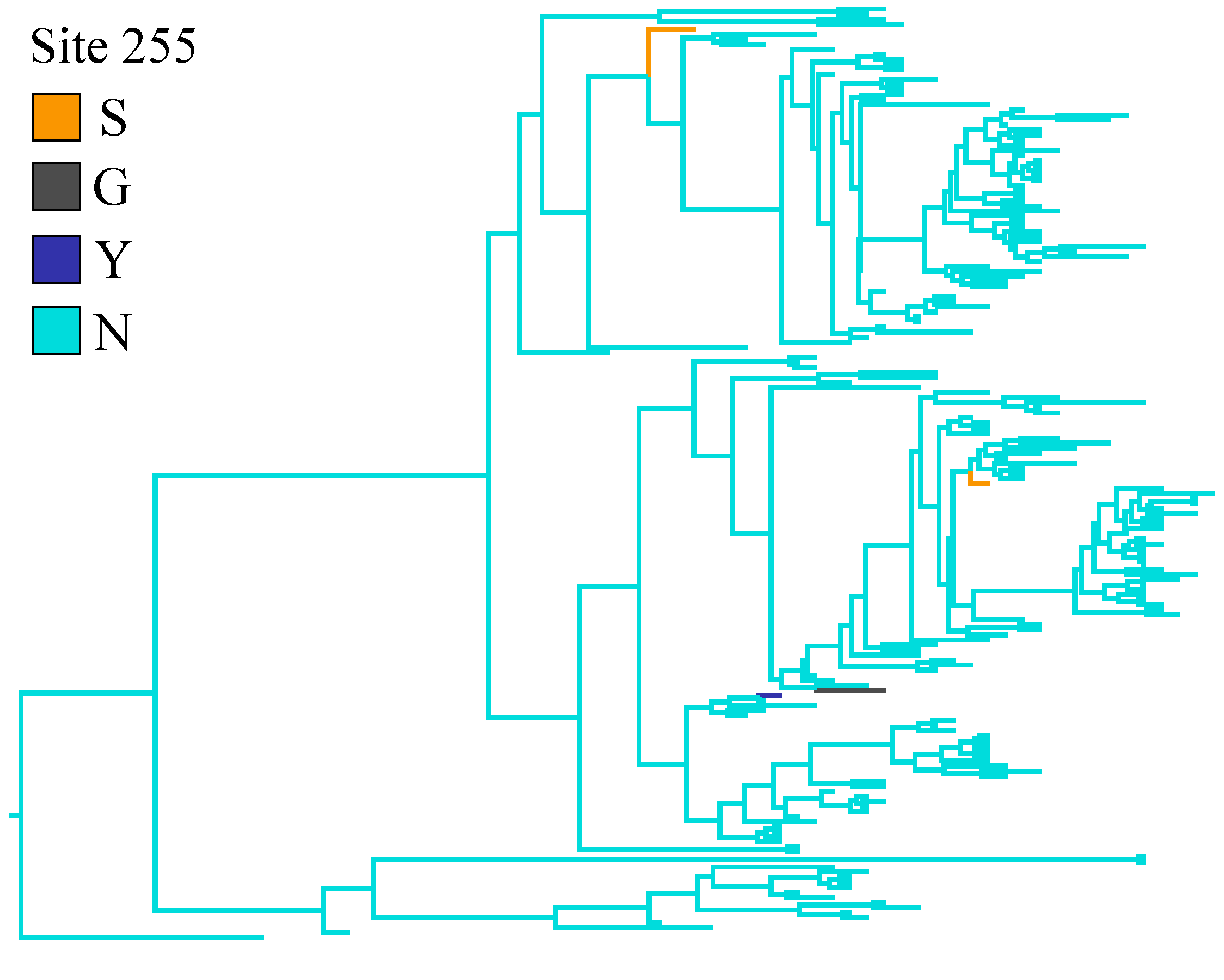

Supplement: Figure S6 — Pervasive and episodic selection sites in the G gene. The G gene based phylogenetic trees show the substitution history for episodic sites 154 and 255 plus the pervasive sites 274 and 290. (ZIP) [file pone.0051439.s006.zip › Figure S6 site 255.tiff]

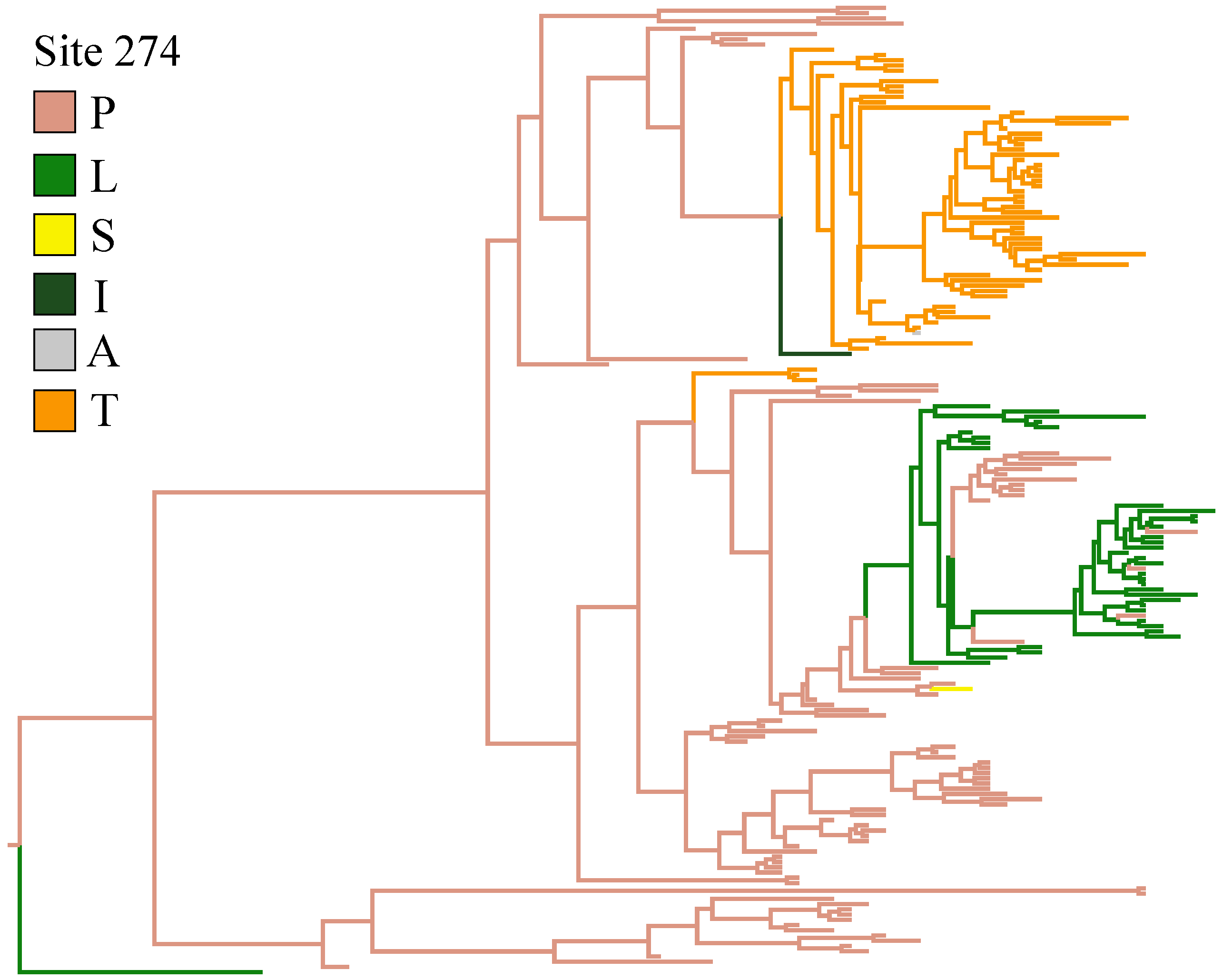

Supplement: Figure S6 — Pervasive and episodic selection sites in the G gene. The G gene based phylogenetic trees show the substitution history for episodic sites 154 and 255 plus the pervasive sites 274 and 290. (ZIP) [file pone.0051439.s006.zip › Figure S6 site 274.tiff]

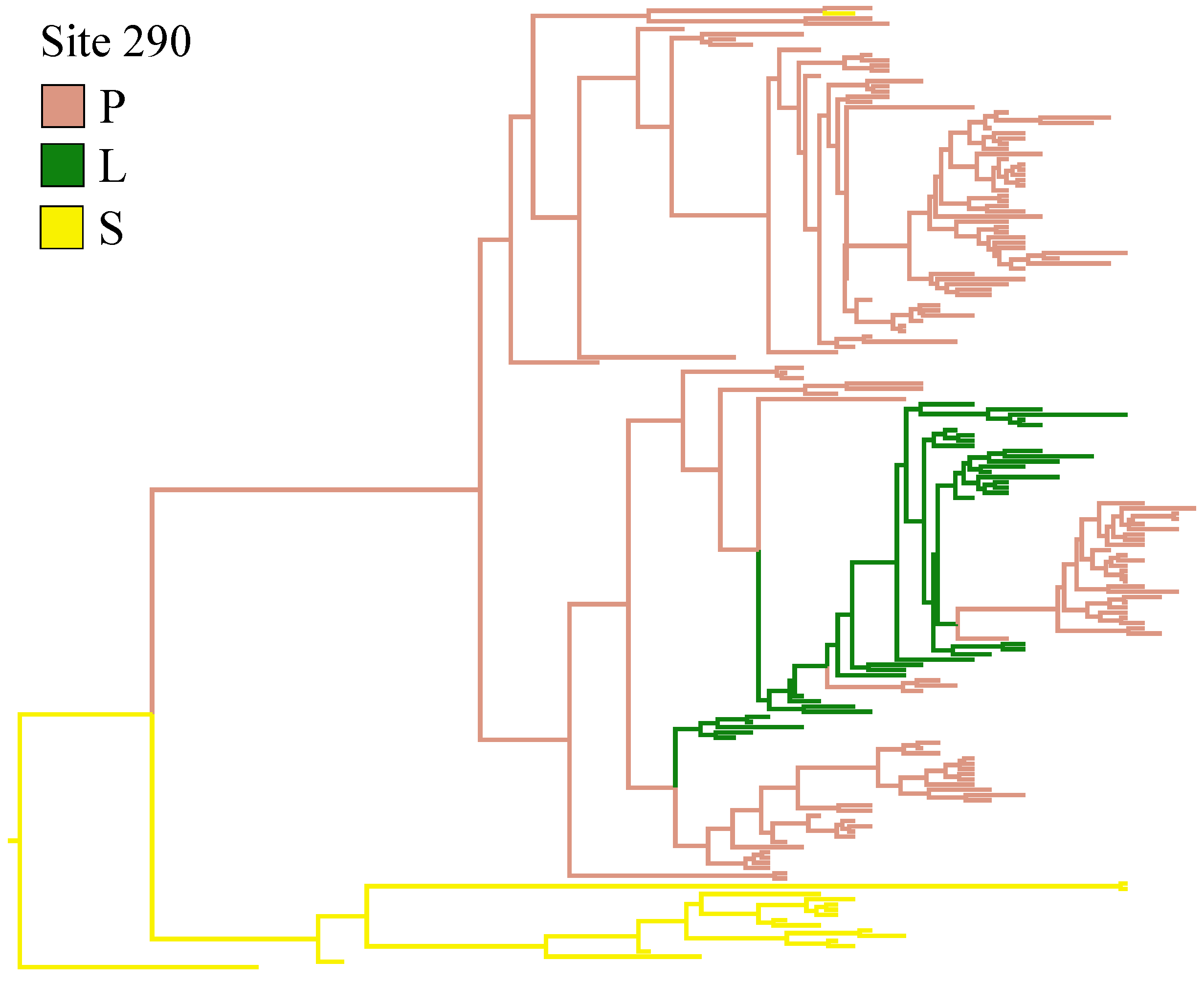

Supplement: Figure S6 — Pervasive and episodic selection sites in the G gene. The G gene based phylogenetic trees show the substitution history for episodic sites 154 and 255 plus the pervasive sites 274 and 290. (ZIP) [file pone.0051439.s006.zip › Figure S6 site 290.tiff]
