# Supplementary material for: Genetic Variability among Complete Human Respiratory Syncytial Virus Subgroup A Genomes: Bridging Molecular Evolutionary Dynamics and Epidemiology
Source: PLoS One. 2012 Dec 7;7(12):e51439. doi: 10.1371/journal.pone.0051439 (PMC3517519; doi:10.1371/journal.pone.0051439)
Supplement: Table S1 — Patient and sample information. (DOC) [file pone.0051439.s007.doc]

| **Code** | **Gender** | **Age (months)*** | **Sample date** | **Material** | **Sequence Method** | **Genotype** | **Accession #** |
| --- | --- | --- | --- | --- | --- | --- | --- |
| 01-000312 | M | 5.7 | 03-01-01 | N Asp | Conventional | GA2 | JQ901447 |
| 01-000583 | V | 0.8 | 07-01-01 | N Asp | Conventional | GA5 | JQ901448 |
| 01-000868 | M | 3.5 | 10-01-01 | N Asp | Conventional | GA5 | JQ901449 |
| 01-002215 | M | 1.4 | 24-01-01 | N Asp | Conventional | GA5 | JQ901450 |
| 01-002279 | M | 3.9 | 24-01-01 | N swab | Conventional | GA5 | JQ901451 |
| 01-031282 | V | 13.4 | 22-12-01 | N Asp | Conventional | GA2 | JQ901452 |
| 02-000110 | V | 3.7 | 03-01-02 | N Asp | Conventional | GA2 | JQ901453 |
| 02-000291 | M | 4.3 | 04-01-02 | N Asp | Conventional | GA5 | JQ901454 |
| 02-017863 | M | 2.1 | 29-06-02 | N Asp | Conventional | GA5 | JQ901455 |
| 03-033338 | M | 3.1 | 25-11-03 | Sputum | Conventional | GA5 | JQ901456 |
| 03-036456 | M | 1.3 | 22-12-03 | N Asp | Conventional | GA2 | JQ901457 |
| 03-036544 | M | 7.9 | 23-12-03 | N Asp | Conventional | GA2 | JQ901458 |
| 05-000257 | M | 1.2 | 04-01-05 | N Asp | Conventional | GA5 | JX015485 |
| 05-000417 | M | 3.0 | 05-01-05 | N Asp | Conventional | GA2 | JX015486 |
| 06-000103 | M | 48.9 | 02-01-06 | N Asp | Conventional | GA5 | JX015487 |
| 06-000827 | V | 10.4 | 09-01-06 | Sputum | Conventional | GA5 | JX015488 |
| 07-039193 | V | 0.5 | 03-12-07 | N Asp | 454, Conventional | GA2 | JX015489 |
| 07-040054 | V | 4.8 | 10-12-07 | N Asp | 454, Conventional | GA2 | JX015480 |
| 07-041785 | M | 3.4 | 24-12-07 | N Asp | 454, Conventional | GA2 | JX015492 |
| 08-000507 | M | 1.0 | 07-01-08 | N Asp | 454 | GA2 | JX015493 |
| 08-001411 | V | 4.2 | 14-01-08 | N Asp | 454 | GA2 | JX015494 |
| 08-042544 | V | 1.6 | 21-11-08 | N Asp | 454 | GA2 | JX015495 |
| 08-042735 | M | 0.4 | 26-11-08 | N Asp | 454, Conventional | GA2 | JX015496 |
| 08-044640 | M | 2.7 | 06-12-08 | N Asp | 454, Conventional | GA2 | JX015497 |
| 08-046972 | M | 1.6 | 27-12-08 | N Asp | 454 | GA2 | JX015498 |
| 08-047045 | M | 2.2 | 29-12-08 | T Asp | 454, Conventional | GA2 | JX015483 |
| RSV572 | M | 10.8 | 06-11-07 | N swab | Conventional | GA2 | JX015484 |
| RSV597 | F | 1.0 | 19-11-07 | N swab | Conventional | GA2 | JX015490 |
| RSV607 | F | 8.1 | 23-11-07 | N swab | Conventional | GA2 | JX015491 |
| 09-000457 | M | 2.1 | 07-01-09 | N Asp | 454, Conventional | GA2 | JX015481 |
| 11-000271 | V | 1.9 | 03-01-11 | N Asp | Conventional | GA2 | JX015479 |
| BE-6650-06 | V | 36.0 | 22-11-06 | N Asp | Conventional | GA2 | JX015482 |
| BE-5146-08 | V | 12.0 | 22-10-08 | N Asp | Conventional | GA2 | JX015499 |
