# Supplementary material for: Genetic Variability among Complete Human Respiratory Syncytial Virus Subgroup A Genomes: Bridging Molecular Evolutionary Dynamics and Epidemiology
Source: PLoS One. 2012 Dec 7;7(12):e51439. doi: 10.1371/journal.pone.0051439 (PMC3517519; doi:10.1371/journal.pone.0051439)
Supplement: Table S2 — Primers used for cDNA preparation and whole genome sequencing. (DOC) [file pone.0051439.s008.doc]

| **Primer** | **5’-3’ sequence** | **Start** | **End** | **Origin** |
| --- | --- | --- | --- | --- |
| 110984SP-1F | ACGCGAAAAAATGCGTACAAC | 1 | 21 | Modified from Kumaria *et al* |
| 110983SP-1R | TGGATGGTGTATTTGCTGGA | 1197 | 1216 | Modified from Kumaria et al |
| 110982SP-2F | ATTTCAACACAAKAKTCACACAA | 1003 | 1025 | Modified from Kumaria et al |
| 110981SP-2R | TTCAGGAGCAAACTTTTCCAT | 2346 | 2366 | Modified from Kumaria et al |
| 111056SP-F2A for | CCATAGTCCAAATGGAGCCTG | 1058 | 1078 | This study |
| 111061SP-F2A rev | CAACTCTACTGCCACCTCTGG | 1837 | 1857 | This study |
| 110980SP-3F | AAAAATTGGGTGGWGAAGCA | 2014 | 2033 | Modified from Kumaria et al |
| 110979SP-3R | CCCTTGGGTGTGGATATTTG | 3441 | 3460 | Modified from Kumaria et al |
| 111057SP-F3A for | GCAGAAGAACTAGAGGCTATC | 2253 | 2273 | This study |
| 111062SP-F3A rev | GGTTGGATGGGTGAGTTGG | 3127 | 3145 | This study |
| 110978SP-4F | AACCTRTTGGAAGGGAATGA | 3018 | 3037 | Modified from Kumaria et al |
| 110977SP-4R | AGGCCAGAATTTGCTTGAGA | 4331 | 4350 | Modified from Kumaria et al |
| 111175F4b-for | GAAGCTATGGCAAGGCTCAG | 2922 | 2941 | This study |
| 111176F4b-rev | CCTTGATTTCGCAGGGTGTG | 3581 | 3600 | This study |
| 111058SP-F5A for | CAGATCATCCCAAGTCATTG | 4153 | 4172 | This study |
| 111063SP-F5A rev | GCTGCATATGCTGCAGGGTAC | 5198 | 5218 | This study |
| 110974SP-6F | AAGTCAACCCTGCAATCCAC | 5054 | 5073 | Modified from Kumaria et al |
| 111167SP-F6a for | CACCATACTAGCTTCAACAACACC | 5023 | 5046 | This study |
| 110973SP-6R | GCATTAACACTAAATTCCCTGGT | 6360 | 6382 | Modified from Kumaria et al |
| 111059SP-F7A for | GTGAACAAGCAAAGCTGC | 6279 | 6296 | This study |
| 111064SP-F7A rev | GTGTGACTGGTGTGCTTCTGG | 7315 | 7335 | This study |
| 110969SP-8F | CCCATTAGTRTTCCCCTCTG | 7097 | 7116 | Modified from Kumaria et al |
| 110968SP-8R | TCCATTAATAATGGGATCCATT | 8497 | 8518 | Modified from Kumaria et al |
| 111060SP-F9A for | GATTGCCAGCAGACGTATTGAAG | 8057 | 8079 | This study |
| 111065SP-F9A rev | GGCTAATATCTTTCCATGTC | 9307 | 9326 | This study |
| 110965SP-10F | CAATGCAACATCCTCCATCA | 9084 | 9103 | Modified from Kumaria et al |
| 110964SP-10R | GGTTGCATTGCAAACATTCTA | 10375 | 10395 | Modified from Kumaria et al |
| 110963SP-11F | CGTGAGTTTCGGTTGCCTA | 10064 | 10082 | Modified from Kumaria et al |
| 110962SP-11R | GGGATCACCACCACCAAATA | 11448 | 11467 | Modified from Kumaria et al |
| 110961SP-12F | AGTGGGACCGTGGATAAACA | 11164 | 11183 | Modified from Kumaria et al |
| 110960SP-12R | TGACTGTAAGGCGATGCAA | 12506 | 12524 | Modified from Kumaria et al |
| 110959SP-13F | TGGACATCAAATATACWACAAGCA | 12180 | 12203 | Modified from Kumaria et al |
| 110958SP-13R | TTAACAACCCAAGGGCAAAC | 13361 | 13380 | Modified from Kumaria et al |
| 110957SP-14F | AAAAAGATTGGGGAGAGGGATA | 13041 | 13062 | Modified from Kumaria et al |
| 110956SP-14R | TGCAYTTTCTTACATGCTTGC | 14355 | 14375 | Modified from Kumaria et al |
| 110955SP-15F | GGTGAAGGAGCAGGGAATTT | 14054 | 14073 | Modified from Kumaria et al |
| 110954SP-15R | ACGAGAAAAAAAGTGTCAAAAACT | 15199 | 15222 | Modified from Kumaria et al |
| 111067F1-seqbeginrev | CTGGCATTGTTGTGAAATTGG | 322 | 342 | This study |
| 111066F15-seqendfor | CCCATAGCTACACACTAAC | 15022 | 15040 | This study |
| 110951Seq-F: | GCAGTCAACCATATGCCTTG |  |  | This study |
| 110952Seq-R | GCTTGCGGAATTCTGACAAC |  |  | This study |
