# Supplementary material for: Genetic Variability among Complete Human Respiratory Syncytial Virus Subgroup A Genomes: Bridging Molecular Evolutionary Dynamics and Epidemiology
Source: PLoS One. 2012 Dec 7;7(12):e51439. doi: 10.1371/journal.pone.0051439 (PMC3517519; doi:10.1371/journal.pone.0051439)
Supplement: Table S3 — Genbank human RSV-A sequences used in this study. (DOC) [file pone.0051439.s009.doc]

| Strain | Accesion No | Country of isolation | Year of isolation | Reference |
| --- | --- | --- | --- | --- |
| USALongs56 | M17212 | U.S.A. | 1956 | Johnson *et al* (1987) PNAS |
| AUSA2s61 | M11486 | Australia | 1961 | Wertz *et al* (1985) PNAS |
| S2s76 | U39662 | U.K. | 1976 | Tolley *et al* (1996) Vaccine |
| 'A/WI/629-3248/98' | JF920062 | U.S.A. | 1998 | Rebuffo-Scheer *et al* (2011) PLoSONE |
| 'A/WI/629-3734/98' | JF920059 | U.S.A. | 1998 | Rebuffo-Scheer *et al* (2011) PLoSONE |
| 'A/WI/629-3868/98' | JF920063 | U.S.A. | 1998 | Rebuffo-Scheer *et al* (2011) PLoSONE |
| 'A/WI/629-4071/98' | JF920065 | U.S.A. | 1998 | Rebuffo-Scheer *et al* (2011) PLoSONE |
| 'A/WI/629-4110/98' | JF920056 | U.S.A. | 1998 | Rebuffo-Scheer *et al* (2011) PLoSONE |
| 'A/WI/629-4239/98' | JF920057 | U.S.A. | 1998 | Rebuffo-Scheer *et al* (2011) PLoSONE |
| 'A/WI/629-4255/98' | JF920068 | U.S.A. | 1998 | Rebuffo-Scheer *et al* (2011) PLoSONE |
| 'A/WI/629-4266/98' | JF920064 | U.S.A. | 1998 | Rebuffo-Scheer *et al* (2011) PLoSONE |
| 'A/WI/629-4285/98' | JF920061 | U.S.A. | 1998 | Rebuffo-Scheer *et al* (2011) PLoSONE |
| 'A/WI/629-4302/98' | JF920067 | U.S.A. | 1998 | Rebuffo-Scheer *et al* (2011) PLoSONE |
| 'A/WI/629-3/06-07' | JF920069 | U.S.A. | 2006-7 | Rebuffo-Scheer *et al* (2011) PLoSONE |
| 'A/WI/629-9/06-07' | JF920070 | U.S.A. | 2006-7 | Rebuffo-Scheer *et al* (2011) PLoSONE |
| 'A/WI/629-17/06-07' | JF920058 | U.S.A. | 2006-7 | Rebuffo-Scheer *et al* (2011) PLoSONE |
| 'A/WI/629-2/07' | JF920046 | U.S.A. | 2007 | Rebuffo-Scheer *et al* (2011) PLoSONE |
| 'A/WI/629-9-2/07' | JF920048 | U.S.A. | 2007 | Rebuffo-Scheer *et al* (2011) PLoSONE |
| 'A/WI/629-21/07' | JF920051 | U.S.A. | 2007 | Rebuffo-Scheer *et al* (2011) PLoSONE |
| 'A/WI/629-22/07' | JF920049 | U.S.A. | 2007 | Rebuffo-Scheer *et al* (2011) PLoSONE |
| 'A/WI/629-23/08' | JF920047 | U.S.A. | 2008 | Rebuffo-Scheer *et al* (2011) PLoSONE |
| 'A/WI/629-DC9/08-09' | JF920050 | U.S.A. | 2008-9 | Rebuffo-Scheer *et al* (2011) PLoSONE |
| 'A/WI/629-Q0154/10' | JF920052 | U.S.A. | 2010 | Rebuffo-Scheer *et al* (2011) PLoSONE |
| 'A/WI/629-Q0198/10' | JF920055 | U.S.A. | 2010 | Rebuffo-Scheer *et al* (2011) PLoSONE |
| 'A/WI/629-Q0282/10' | JF920054 | U.S.A. | 2010 | Rebuffo-Scheer *et al* (2011) PLoSONE |
| 'A/WI/629-Q0284/10' | JF920053 | U.S.A. | 2010 | Rebuffo-Scheer *et al* (2011) PLoSONE |
| BE156s84 | AY343597 | Belgium | 1984 | Zlateva *et al* (2004) J Virol |
| BE204s84 | AY343625 | Belgium | 1984 | Zlateva *et al* (2004) J Virol |
| BE183s85 | AY343626 | Belgium | 1985 | Zlateva *et al* (2004) J Virol |
| BE2466s85 | AY343631 | Belgium | 1985 | Zlateva *et al* (2004) J Virol |
| BE2584s85 | AY343596 | Belgium | 1985 | Zlateva *et al* (2004) J Virol |
| BE76s86 | AY343632 | Belgium | 1986 | Zlateva *et al* (2004) J Virol |
| BE339s86 | AY343633 | Belgium | 1986 | Zlateva *et al* (2004) J Virol |
| BE410s86 | AY343640 | Belgium | 1986 | Zlateva *et al* (2004) J Virol |
| BE3252s86 | AY343641 | Belgium | 1986 | Zlateva *et al* (2004) J Virol |
| BE119s87 | AY343656 | Belgium | 1987 | Zlateva *et al* (2004) J Virol |
| BE307s87 | AY343655 | Belgium | 1987 | Zlateva *et al* (2004) J Virol |
| BE3785s87 | AY343654 | Belgium | 1987 | Zlateva *et al* (2004) J Virol |
| BE4147s87 | AY343622 | Belgium | 1987 | Zlateva *et al* (2004) J Virol |
| BE071s88 | AY343623 | Belgium | 1988 | Zlateva *et al* (2004) J Virol |
| BE538s88 | AY343624 | Belgium | 1988 | Zlateva *et al* (2004) J Virol |
| BE933s88 | AY343595 | Belgium | 1988 | Zlateva *et al* (2004) J Virol |
| BE4763s88 | AY343628 | Belgium | 1988 | Zlateva *et al* (2004) J Virol |
| BE305s89 | AY343653 | Belgium | 1989 | Zlateva *et al* (2004) J Virol |
| BE1587s89 | AY343587 | Belgium | 1989 | Zlateva *et al* (2004) J Virol |
| BE1591s90 | AY343620 | Belgium | 1990 | Zlateva *et al* (2004) J Virol |
| BE191s90 | AY343659 | Belgium | 1990 | Zlateva *et al* (2004) J Virol |
| BE138s90 | AY343658 | Belgium | 1990 | Zlateva *et al* (2004) J Virol |
| BE369s90 | AY343660 | Belgium | 1990 | Zlateva *et al* (2004) J Virol |
| BE6274s91 | AY343649 | Belgium | 1991 | Zlateva *et al* (2004) J Virol |
| BE6460s91 | AY343652 | Belgium | 1991 | Zlateva *et al* (2004) J Virol |
| BE1440s92 | AY343651 | Belgium | 1992 | Zlateva *et al* (2004) J Virol |
| BE8078s92 | AY343657 | Belgium | 1992 | Zlateva *et al* (2004) J Virol |
| BE614s93 | AY343586 | Belgium | 1993 | Zlateva *et al* (2004) J Virol |
| BE10490s93 | AY343584 | Belgium | 1993 | Zlateva *et al* (2004) J Virol |
| BE11465s94 | AY343615 | Belgium | 1994 | Zlateva *et al* (2004) J Virol |
| BE11600s94 | AY343588 | Belgium | 1994 | Zlateva *et al* (2004) J Virol |
| BE12005s94 | AY343590 | Belgium | 1994 | Zlateva *et al* (2004) J Virol |
| BE174s95 | AY343591 | Belgium | 1995 | Zlateva *et al* (2004) J Virol |
| BE521s95 | AY343621 | Belgium | 1995 | Zlateva *et al* (2004) J Virol |
| BE12895s95 | AY343619 | Belgium | 1995 | Zlateva *et al* (2004) J Virol |
| BE13551s95 | AY343618 | Belgium | 1995 | Zlateva *et al* (2004) J Virol |
| BE12216s96 | AY343592 | Belgium | 1996 | Zlateva *et al* (2004) J Virol |
| BE12243s96 | AY343647 | Belgium | 1996 | Zlateva *et al* (2004) J Virol |
| BE12350s96 | AY343593 | Belgium | 1996 | Zlateva *et al* (2004) J Virol |
| BE15471s97 | AY343594 | Belgium | 1997 | Zlateva *et al* (2004) J Virol |
| BE15752s97 | AY343617 | Belgium | 1997 | Zlateva *et al* (2004) J Virol |
| BE14461s98 | AY343614 | Belgium | 1998 | Zlateva *et al* (2004) J Virol |
| Strain | Accesion No | Country of isolation | Year of isolation | Reference |
| BE14536s98 | AY343583 | Belgium | 1998 | Zlateva et al (2004) J Virol |
| BE14808s98 | AY343609 | Belgium | 1998 | Zlateva et al (2004) J Virol |
| BE14898s98 | AY343610 | Belgium | 1998 | Zlateva et al (2004) J Virol |
| BE13192s99 | AY343556 | Belgium | 1999 | Zlateva et al (2004) J Virol |
| BE13280s99 | AY343554 | Belgium | 1999 | Zlateva et al (2004) J Virol |
| BE13281s99 | AY343568 | Belgium | 1999 | Zlateva et al (2004) J Virol |
| BE13393s99 | AY343612 | Belgium | 1999 | Zlateva et al (2004) J Virol |
| BE13412s99 | AY343646 | Belgium | 1999 | Zlateva et al (2004) J Virol |
| BE13425s99 | AY343603 | Belgium | 1999 | Zlateva et al (2004) J Virol |
| BE13462s99 | AY343643 | Belgium | 1999 | Zlateva et al (2004) J Virol |
| BE16s100 | AY343558 | Belgium | 2000 | Zlateva et al (2004) J Virol |
| BE797s100 | AY343581 | Belgium | 2000 | Zlateva et al (2004) J Virol |
| BE800s100 | AY343561 | Belgium | 2000 | Zlateva et al (2004) J Virol |
| BE822s100 | AY343567 | Belgium | 2000 | Zlateva et al (2004) J Virol |
| BE944s100 | AY343582 | Belgium | 2000 | Zlateva et al (2004) J Virol |
| BE1061s100 | AY343557 | Belgium | 2000 | Zlateva et al (2004) J Virol |
| BE1150s100 | AY343579 | Belgium | 2000 | Zlateva et al (2004) J Virol |
| BE1936s100 | AY343569 | Belgium | 2000 | Zlateva et al (2004) J Virol |
| BE1937s100 | AY343572 | Belgium | 2000 | Zlateva et al (2004) J Virol |
| BE2122s100 | AY343559 | Belgium | 2000 | Zlateva et al (2004) J Virol |
| BE2149s100 | AY343573 | Belgium | 2000 | Zlateva et al (2004) J Virol |
| BE11030s100 | AY343599 | Belgium | 2000 | Zlateva et al (2004) J Virol |
| BE11091s100 | AY343601 | Belgium | 2000 | Zlateva et al (2004) J Virol |
| BE11129s100 | AY343600 | Belgium | 2000 | Zlateva et al (2004) J Virol |
| BE11976s100 | AY343565 | Belgium | 2000 | Zlateva et al (2004) J Virol |
| BE12028s100 | AY343608 | Belgium | 2000 | Zlateva et al (2004) J Virol |
| BE11s101 | AY343611 | Belgium | 2001 | Zlateva et al (2004) J Virol |
| BE64s101 | AY343566 | Belgium | 2001 | Zlateva et al (2004) J Virol |
| BE112s101 | AY343578 | Belgium | 2001 | Zlateva et al (2004) J Virol |
| BE519s101 | AY343605 | Belgium | 2001 | Zlateva et al (2004) J Virol |
| BE1441s101 | AY343562 | Belgium | 2001 | Zlateva et al (2004) J Virol |
| BE1224s101 | AY343571 | Belgium | 2001 | Zlateva et al (2004) J Virol |
| BE1343s101 | AY343574 | Belgium | 2001 | Zlateva et al (2004) J Virol |
| BE1556s101 | AY343560 | Belgium | 2001 | Zlateva et al (2004) J Virol |
| BE1717s101 | AY343577 | Belgium | 2001 | Zlateva et al (2004) J Virol |
| BE1835s101 | AY343570 | Belgium | 2001 | Zlateva et al (2004) J Virol |
| BE1836s101 | AY343576 | Belgium | 2001 | Zlateva et al (2004) J Virol |
| BE11584s101 | AY343645 | Belgium | 2001 | Zlateva et al (2004) J Virol |
| BE004s102 | AY343606 | Belgium | 2002 | Zlateva et al (2004) J Virol |
| BE332s102 | AY343613 | Belgium | 2002 | Zlateva et al (2004) J Virol |
| BIR642s89 | X73354 | U.K. | 1989 | Cane et al (1994) J Clin Micro |
| BIR6190s89 | X73350 | U.K. | 1989 | Cane et al (1994) J Clin Micro |
| BIR1734s89 | X73352 | U.K. | 1989 | Cane et al (1994) J Clin Micro |
| MADs91 | Z33420 | Spain | 1991 | Garcia et al (1994) J Virol |
| MAD6s92 | Z33418 | Spain | 1992 | Garcia et al (1994) J Virol |
| MAD8s92 | Z33419 | Spain | 1992 | Garcia et al (1994) J Virol |
| MAD1s93 | Z33414 | Spain | 1993 | Garcia et al (1994) J Virol |
| MAD2s93 | Z33493 | Spain | 1993 | Garcia et al (1994) J Virol |
| MAD6s93 | Z33410 | Spain | 1993 | Garcia et al (1994) J Virol |
| MON2s88 | Z33424 | Uruguay | 1988 | Garcia et al (1994) J Virol |
| MON1s89 | Z33422 | Uruguay | 1989 | Garcia et al (1994) J Virol |
| MON1s90 | Z33494 | Uruguay | 1990 | Garcia et al (1994) J Virol |
| MON5s90 | Z33427 | Uruguay | 1990 | Garcia et al (1994) J Virol |
| MON5s91 | Z33428 | Uruguay | 1991 | Garcia et al (1994) J Virol |
| MON9s91 | Z33431 | Uruguay | 1991 | Garcia et al (1994) J Virol |
| MON1s92 | Z33423 | Uruguay | 1992 | Garcia et al (1994) J Virol |
| MON9s92 | Z33432 | Uruguay | 1992 | Garcia et al (1994) J Virol |
| NYCH09s93 | AF065254 | U.S.A. | 1993 | Peret et al (1996) J Gen Virol |
| NYCH17s93 | AF065255 | U.S.A. | 1993 | Peret et al (1996) J Gen Virol |
| NYCH34s94 | AF065257 | U.S.A. | 1994 | Peret et al (1996) J Gen Virol |
| NYCH57s94 | AF065258 | U.S.A. | 1994 | Peret et al (1996) J Gen Virol |
| SE03s91 | AF193304 | Korea | 1991 | Choi et al (2000) J Infect Dis |
| SE05s91 | AF193306 | Korea | 1991 | Choi et al (2000) J Infect Dis |
| SE10s91 | AF193307 | Korea | 1992 | Choi et al (2000) J Infect Dis |
| SE01s92 | AF193308 | Korea | 1992 | Choi et al (2000) J Infect Dis |
| SE09s92 | AF193309 | Korea | 1992 | Choi et al (2000) J Infect Dis |
| SE10s92 | AF193310 | Korea | 1992 | Choi et al (2000) J Infect Dis |
| SE11s92 | AF193311 | Korea | 1992 | Choi et al (2000) J Infect Dis |

| Strain | Accesion No | Country of isolation | Year of isolation | Reference |
| --- | --- | --- | --- | --- |
| SE12s92 | AF193312 | Korea | 1992 | Choi et al (2000) J Infect Dis |
| SE12s94 | AF193316 | Korea | 1994 | Choi *et al* (2000) J Infect Dis |
| SE01s95 | AF193317 | Korea | 1995 | Choi *et al* (2000) J Infect Dis |
| SE11s95 | AF193318 | Korea | 1995 | Choi *et al* (2000) J Infect Dis |
| SE12s95 | AF193319 | Korea | 1995 | Choi *et al* (2000) J Infect Dis |
| SE05s96 | AF193321 | Korea | 1996 | Choi *et al* (2000) J Infect Dis |
| SE08s96 | AF193322 | Korea | 1996 | Choi *et al* (2000) J Infect Dis |
| SE12s97 | AF193323 | Korea | 1997 | Choi *et al* (2000) J Infect Dis |
| SE02s98 | AF193325 | Korea | 1998 | Choi *et al* (2000) J Infect Dis |
| WV2780s79 | AF065405 | U.S.A. | 1979 | Sullender *et al* (1991) J Clin Micro |
| WV5222s81 | AF065406 | U.S.A. | 1981 | Sullender *et al* (1991) J Clin Micro |
| WV6973s82 | AF065407 | U.S.A. | 1982 | Sullender *et al* (1991) J Clin Micro |
| WV12342s84 | AF065409 | U.S.A. | 1984 | Sullender *et al* (1991) J Clin Micro |
| WV19983s87 | AF065408 | U.S.A. | 1987 | Sullender *et al* (1991) J Clin Micro |
| WV23836s88 | AF065410 | U.S.A. | 1988 | Sullender *et al* (1991) J Clin Micro |
