# Supplementary material for: Genetic Variability among Complete Human Respiratory Syncytial Virus Subgroup A Genomes: Bridging Molecular Evolutionary Dynamics and Epidemiology
Source: PLoS One. 2012 Dec 7;7(12):e51439. doi: 10.1371/journal.pone.0051439 (PMC3517519; doi:10.1371/journal.pone.0051439)
Supplement: Table S4 — Overview protein sequence variability per RSV strain. (DOC) [file pone.0051439.s010.doc]

|  | NS1 | NS2 | N | P | M | SH | G | F | M2-1 | M2-2 | L |
| --- | --- | --- | --- | --- | --- | --- | --- | --- | --- | --- | --- |
| Consensus | - | - | - | - | - | - | - | - | - | - | - |
| 01-000312 | 6 | 8 | 7 | 5 | 6 | 10 | 14 | 6 | 5 | 9 | 5 |
| 01-000583 | 6 | 8 | 7 | 5 | 6 | 8 | 18 | 7 | 5 | 10 | 5 |
| 01-000868 | 6 | 8 | 8 | 5 | 6 | 8 | 17 | 7 | 5 | 10 | 5 |
| 01-002215 | 6 | 8 | 7 | 5 | 6 | 8 | 17 | 7 | 5 | 12 | 5 |
| 01-002279 | 6 | 8 | 7 | 5 | 6 | 8 | 16 | 7 | 5 | 13 | 5 |
| 01-031282 | 6 | 7 | 7 | 5 | 6 | 8 | 13 | 7 | 5 | 10 | 5 |
| 02-000110 | 6 | 7 | 7 | 5 | 6 | 8 | 13 | 7 | 5 | 10 | 5 |
| 02-000291 | 6 | 8 | 8 | 5 | 6 | 8 | 16 | 7 | 6 | 10 | 5 |
| 02-017863 | 6 | 8 | 7 | 5 | 6 | 8 | 18 | 7 | 5 | 12 | 5 |
| 03-033338 | 6 | 8 | 8 | 5 | 6 | 8 | 16 | 7 | 6 | 10 | 5 |
| 03-036456 | 6 | 8 | 7 | 5 | 6 | 8 | 14 | 6 | 5 | 12 | 5 |
| 03-036544 | 6 | 7 | 7 | 5 | 6 | 8 | 13 | 7 | 6 | 9 | 5 |
| 05-000257 | 6 | 8 | 7 | 5 | 6 | 8 | 16 | 7 | 6 | 12 | 5 |
| 05-000417 | 6 | 7 | 7 | 5 | 6 | 8 | 13 | 7 | 6 | 10 | 5 |
| 06-000103 | 6 | 8 | 7 | 5 | 6 | 8 | 17 | 7 | 5 | 10 | 5 |
| 06-000827 | 6 | 8 | 7 | 5 | 6 | 8 | 17 | 7 | 6 | 12 | 5 |
| 07-039193 | 6 | 7 | 7 | 5 | 6 | 8 | 11 | 6 | 5 | 9 | 5 |
| 07-040054 | 6 | 7 | 7 | 5 | 6 | 8 | 14 | 7 | 5 | 14 | 5 |
| 07-041785 | 6 | 7 | 7 | 5 | 6 | 8 | 11 | 6 | 5 | 9 | 5 |
| 08-000507 | 6 | 7 | 7 | 6 | 6 | 8 | 11 | 6 | 5 | 9 | 5 |
| 08-001411 | 6 | 7 | 7 | 5 | 6 | 10 | 10 | 6 | 5 | 10 | 5 |
| 08-042544 | 6 | 7 | 8 | 5 | 6 | 8 | 10 | 6 | 5 | 9 | 5 |
| 08-042735 | 6 | 7 | 7 | 5 | 6 | 8 | 11 | 7 | 5 | 9 | 5 |
| 08-044640 | 6 | 7 | 7 | 5 | 6 | 8 | 11 | 6 | 5 | 12 | 5 |
| 08-046972 | 6 | 8 | 7 | 5 | 6 | 8 | 11 | 6 | 5 | 9 | 5 |
| 08-047045 | 6 | 8 | 7 | 5 | 6 | 8 | 10 | 7 | 5 | 9 | 5 |
| 09-000457 | 6 | 7 | 7 | 5 | 6 | 8 | 12 | 6 | 5 | 10 | 5 |
| 11-000271 | 6 | 9 | 7 | 5 | 6 | 10 | 11 | 7 | 5 | 10 | 5 |
| AY911262.1_Long | 6 | 8 | 7 | 6 | 6 | 10 | 18 | 7 | 6 | 20 | 6 |
| BE-5146-08 | 6 | 7 | 7 | 5 | 6 | 8 | 11 | 6 | 5 | 9 | 5 |
| BE-6650-06 | 6 | 7 | 7 | 5 | 6 | 8 | 10 | 6 | 5 | 10 | 5 |
| FJ614813.1_Line_19 | 6 | 8 | 7 | 6 | 6 | 10 | 18 | 8 | 6 | 20 | 6 |
| M74568.1_A2 | 6 | 9 | 7 | 5 | 6 | 8 | 18 | 9 | 5 | 20 | 6 |
| NC_001803.1_RSS-2 | 6 | 8 | 7 | 6 | 6 | 8 | 13 | 7 | 5 | 9 | 5 |
| RSV572 | 6 | 7 | 7 | 5 | 6 | 8 | 11 | 6 | 5 | 9 | 5 |
| RSV597 | 6 | 8 | 7 | 5 | 6 | 8 | 12 | 6 | 5 | 9 | 5 |
| RSV607 | 6 | 7 | 7 | 5 | 6 | 8 | 11 | 6 | 5 | 9 | 5 |
