# Supplementary material for: Genetic Variability among Complete Human Respiratory Syncytial Virus Subgroup A Genomes: Bridging Molecular Evolutionary Dynamics and Epidemiology
Source: PLoS One. 2012 Dec 7;7(12):e51439. doi: 10.1371/journal.pone.0051439 (PMC3517519; doi:10.1371/journal.pone.0051439)
Supplement: Table S5 — N-glycosylation probability report of Asn-Xaa-Ser/Thr sequons in the RSV G protein. (DOC) [file pone.0051439.s011.doc]

| **Position of Asp** | **85** | **103** | **135** | **179** | **237** | **242** | **244** | **250** | **251** | **258** | **273** | **294** |
| --- | --- | --- | --- | --- | --- | --- | --- | --- | --- | --- | --- | --- |
| Consensus | --- | X | + | - | X | X | X | X | + | X | + | - |
| 01-000312 | --- | + | + | - | + | X | X | X | X | X | --- | + |
| 01-000583 | --- | ++ | + | - | + | X | X | + | X | X | --- | + |
| 01-000868 | --- | ++ | + | - | + | X | X | + | X | X | --- | + |
| 01-002215 | --- | ++ | + | - | + | X | X | + | X | X | --- | + |
| 01-002279 | --- | ++ | + | - | + | X | X | + | X | X | --- | + |
| 01-031282 | --- | + | + | - | + | X | X | X | + | X | --- | + |
| 02-000110 | --- | + | + | - | + | X | X | X | + | X | --- | + |
| 02-000291 | --- | ++ | + | - | + | X | X | + | X | X | --- | + |
| 02-017863 | --- | ++ | + | - | + | X | X | + | X | X | --- | + |
| 03-033338 | --- | ++ | + | - | + | X | X | + | X | X | --- | + |
| 03-036456 | --- | + | - | - | + | X | X | X | + | X | --- | + |
| 03-036544 | --- | + | - | - | + | X | X | X | + | X | --- | + |
| 05-000257 | --- | ++ | + | - | + | X | X | + | X | X | --- | + |
| 05-000417 | --- | + | - | - | + | X | - | X | + | X | --- | + |
| 06-000103 | --- | ++ | + | - | + | X | X | + | X | X | --- | + |
| 06-000827 | --- | ++ | + | - | + | X | X | + | X | X | --- | + |
| 07-039193 | --- | + ProX1 | + | - | X | X | X | X | + | X | --- | + |
| 07-040054 | --- | + | --- | - | + | X | X | X | ++ | - | --- | + |
| 07-041785 | --- | + ProX1 | + | - | X | X | X | X | + | X | --- | + |
| 08-000507 | --- | + ProX1 | + | - | X | X | X | X | + | X | --- | + |
| 08-001411 | --- | + | + | - | X | X | X | X | + | X | --- | + |
| 08-042544 | --- | + | + | - | X | X | X | X | + | X | --- | + |
| 08-042735 | --- | + | + | - | X | X | X | X | + | X | --- | + |
| 08-044640 | --- | ++ | + | - | X | X | X | X | - | X | --- | + |
| 08-046972 | --- | + | + | - | X | X | X | X | X | X | --- | + |
| 08-047045 | --- | + | + | - | X | X | X | X | + | X | --- | + |
| 09-000457 | --- | ++ | + | - | X | X | X | X | - | X | --- | + |
| 11-000271 | --- | + | + | - | X | X | X | X | + | X | --- | X |
| AY911262.1_Long | --- | + | + | - | + | + | X | - | + | X | --- | + |
| BE-5146-08 | --- | + | + | - | X | X | X | X | X | X | --- | + |
| BE-6650-06 | --- | + ProX1 | + | - | X | X | X | X | + | X | --- | + |
| FJ614813.1_Line_19 | --- | + | + | - | + | + | X | - | + | X | --- | + |
| M74568.1_A2 | --- | + | - | - | + | X | X | X | + | X | --- | X |
| NC_001803.1_RSS-2 | --- | + | - | - | + | X | X | - | + | X | --- | + |
| RSV572 | --- | + ProX1 | + | - | X | X | X | X | + | X | --- | + |
| RSV597 | --- | + | + | - | X | X | X | X | + | X | --- | - |
| RSV607 | --- | + ProX1 | + | - | X | X | X | X | + | X | --- | + |
