# Supplementary material for: Genetic Variability among Complete Human Respiratory Syncytial Virus Subgroup A Genomes: Bridging Molecular Evolutionary Dynamics and Epidemiology
Source: PLoS One. 2012 Dec 7;7(12):e51439. doi: 10.1371/journal.pone.0051439 (PMC3517519; doi:10.1371/journal.pone.0051439)
Supplement: Table S6 — Clock comparison for marginal likelihood estimates. (DOC) [file pone.0051439.s012.doc]

|  | **Model** | **Strict** | **Relaxed** |
| --- | --- | --- | --- |
| Distributed | HME | **-42070.9306** | -42074.4266 |
|  | AICm | **84186.937** | 84214.735 |
|  | PS | -42371.63785 | **84043.193** |
|  | SS | -42372.11672 | **-42363.75947** |
| Removed | HME | -41994.9657 | **-41983.6427** |
|  | AICm | **84021.414** | 84043.193 |
|  | PS | -42268.32216 | **-42263.49189** |
|  | SS | -42268.73537 | **-42263.95108** |
